# Aromatic Biobased Polymeric Materials Using Plant Polyphenols as Sustainable Alternative Raw Materials: A Review

**DOI:** 10.3390/polym16192752

**Published:** 2024-09-29

**Authors:** Yang Liu, Junsheng Wang, Zhe Sun

**Affiliations:** 1 Tianjin Fire Science and Technology Research Institute of MEM, Tianjin 300381, China; liuyang@tfri.com.cn; 2 Key Laboratory of Fire Protection Technology for Industry and Public Building, Ministry of Emergency Management, Tianjin 300381, China; 3 Tianjin Key Laboratory of Fire Safety Technology, Tianjin 300381, China; 4 College of Textile and Clothing Engineering, Soochow University, Suzhou 215123, China

**Keywords:** plant polyphenols, polymeric materials, sustainability, green chemistry, industrial application

## Abstract

In the foreseeable future, the development of petroleum-based polymeric materials may be limited, owing to the gradual consumption of disposable resources and the increasing emphasis on environmental protection policies. Therefore, it is necessary to focus on introducing environmentally friendly renewable biobased materials as a substitute for petroleum-based feed stocks in the preparation of different types of industrially important polymers. Plant polyphenols, a kind of natural aromatic biomolecule, exist widely in some plant species. Benefiting from their special macromolecular structure, high reactivity, and broad abundance, plant polyphenols are potent candidates to replace the dwindling aromatic monomers derived from petroleum-based resources in synthesizing high-quality polymeric materials. In this review, the most related and innovative methods for elaborating novel polymeric materials from plant polyphenols are addressed. After a brief historical overview, the classification, structural characteristics, and reactivity of plant polyphenols are summarized in detail. In addition, some interesting and innovative works concerning the chemical modifications and polymerization techniques of plant polyphenols are also discussed. Importantly, the main chemical pathways to create plant polyphenol-based organic/organic–inorganic polymeric materials as well as their properties and possible applications are systematically described. We believe that this review could offer helpful references for designing multifunctional polyphenolic materials.

## 1. Introduction

Plant polyphenols (also known as tannins), a category of naturally occurring compounds with multiple phenolic hydroxyl (Ph-OH) groups, can be widely extracted from the bark, needles, fruits, and leaves of a variety of plant species [1,2,3,4,5]. They are one of the most abundant natural compounds obtained from biobased resources [6,7]. In vascular plants, plant polyphenols primarily function as protective agents against light exposure (especially for UV radiation) and defend from biological threats coming from animals, insects, and bacteria [8]. Furthermore, for non-vascular plants (such as algae), in which plant polyphenols possess various metabolic effects [9], including cell wall creation [10], herbivorous organism defense [11], and so on. Usually, the concentration of plant polyphenols in pine bark, mimosa bark, and quebracho wood is relatively high [12,13,14]. It should be mentioned that the plant polyphenol content in plants varies with the weather conditions, soil quality, light time and intensity, etc. [15].

**Figure 1:**
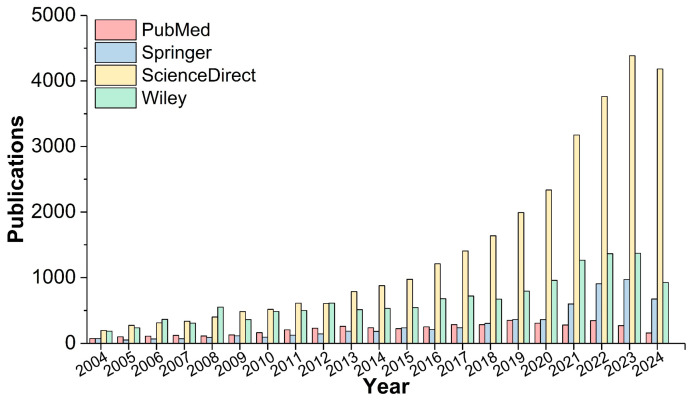
Number of annual publications with the term “plant polyphenols polymerization” from 2004 to 2024. Data were collected from PubMed, Springer, ScienceDirect, and Wiley on 25 July 2024.

Long ago, as a byproduct of biomass resources, plant polyphenols were typically utilized as an energy through incineration. Therefore, the effective and functional utilization of plant polyphenols obtained from various plant species provides a vital and sustainable alternative for enhancing their added value. Originally, plant polyphenols were utilized in leather making as vegetable tanning agents to achieve the conversion of hides into leather products [16,17]. Nowadays, other applications of plant polyphenols can also be seen in more and more fields, such as dyestuff, food, coating, environment, biomedicine, and dentistry [18,19,20,21,22], mainly owing to their high reactivity, good biocompatibility, lower toxicity and cost, antimicrobial activity, and so on. In the past few years, petroleum-based materials have experienced rapid development. However, their fast growth may be inhibited by the fluctuating oil price and increasingly rigorous environmental policies. With the growing demand for sustainable materials, the applications of eco-friendly biomass alternatives (i.e., plant polyphenols) are gaining increasing importance. Plant polyphenols can be easily incorporated into the polymer matrix through physical blending or chemical polymerization, including being copolymerized with isocyanates, epoxy groups, phenol-aldehyde resins, amines, etc., to obtain novel functional polymers or reactive intermediates [23,24,25,26,27,28,29]. In addition, plant polyphenols can also form organic/organic–inorganic nanoparticles or coatings through self-polymerization or complexation reactions with metal ions [30,31]. Altogether, the utilization of plant polyphenols to fabricate biobased polymers/intermediates, or as additives to endow existing polymeric materials with functionality or enhance their inherent properties, has shown significant advantages at both molecular design and environmental levels. Therefore, benefiting from the fascinating and promising properties of polymeric materials endowed by plant polyphenols, the number of studies on plant polyphenol polymerization has shown an increasing trend over the past two decades (Figure 1).

As one of the natural aromatic resources, the Ph-OH groups in plant polyphenols have relatively high reactivity, which can undergo substitution, condensation, coordination reactions, etc. It should be mentioned that these chemical reactions are essential for the creation of elaborated plant polyphenol-based monomers or polymers (i.e., polyurethanes, epoxy resins, polyesters, etc.) with interesting performance. To date, there are many works about the extraction, modification, and characterization of plant polyphenols. However, only a few comprehensive reviews focus on the different kinds of plant polyphenol-derived oligomer/polymers and nanoparticles, lacking an evaluation of their functionality. Therefore, the goal of this work is to furnish a systematic overview of the development, classification and structure, reactivity, and polymerization techniques of plant polyphenols. Importantly, recent attempts for creating plant polyphenol-based organic/organic–inorganic polymeric materials are systematically summarized and discussed, with a particular focus on modification methods, material multifunctionality, and potential application areas.

## 2. Plant Polyphenol Chemistry

### 2.1. Historical Outline

The utilization of plant polyphenols by humans predates understanding. In the beginning, people were able to consciously use plant polyphenols for leather making (achieving the conversion of hide into leather) [15], that is, by soaking animal skins in water with some plants. The systematic study of plant polyphenols began in the late 18th century [11]; before being known as plant polyphenols, those plant-derived natural substances (plant extracts) that can convert animal skins into leather were commonly referred to as “vegetable tannin”, as pointed out by Seguin in 1796. With the further development of polyphenol chemistry, White (1957), Bate-Smith and Swain (1962) defined that the term “vegetable tannin” should strictly refer to water-soluble plant polyphenols with a molecular weight between 500 and 3000 Da. In 1981, this definition was refined at the molecular level by Haslam, who expanded the definitions proposed by White, Bate-Smith and Swain [32]. The term “plant polyphenols” should include tannins and their related derived compounds (such as tannin precursor compounds and tannin polymers), which can comprehensively summarize the characteristics of such natural products. Importantly, this descriptor is also in line with the actual research situation of various disciplines, so it is widely adopted.

### 2.2. Classification and Structure

**Figure 2:**
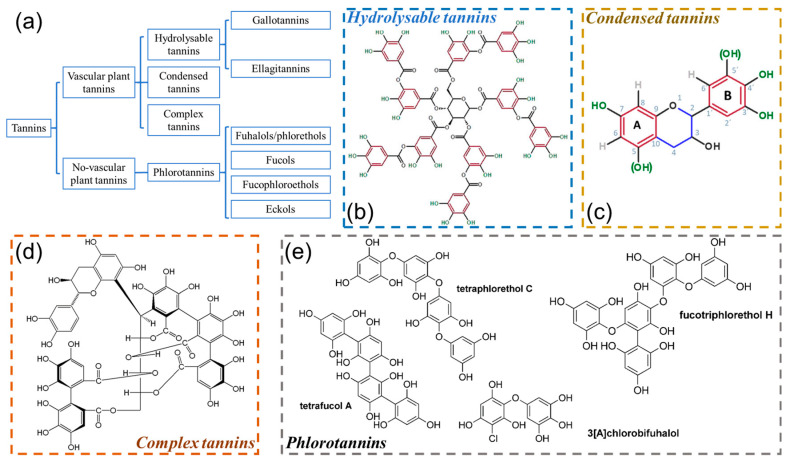
(**a**) Tannins classification. Structures of (**b**) hydrolysable tannins (tannic acid), (**c**) condensed tannins, reproduced from Reference [37] under Creative Commons License 4.0, (**d**) complex tannins, and (**e**) representative examples of phlorotannins, reproduced from Reference [32] with permission of John Wiley and Sons, Copyrights (2011).

Plant polyphenols are a kind of secondary metabolites with a phenolic structure and widely exist in the skin, roots, needles, and fruits of plants [33,34]. They are currently divided into the following four categories (Figure 2a): hydrolysable tannins, condensed tannins, complex tannins, and phlorotannins.

#### 2.2.1. Hydrolysable Tannins

Hydrolysable tannins are considered to be polyester compounds consisting of organic acids and sugar units (tannic acid as a typical representative, Figure 2b), which can be further divided into two classes: the gallo- and ellagitannins. As the name of hydrolysable tannins demonstrates, this type of tannin is easily hydrolyzed via weak bases or acidic media [35,36]. Specifically, gallic acid (and derivatives) can be obtained via the hydrolysis of gallotannins; ellagic acid can be generated via the hydrolysis of ellagitannins [37]. These hydrolysable tannins are mainly applied in the leather industry for tanning as they can endow leather products with desirable clarity and light resistance [35], but the worldwide production is relatively low.

#### 2.2.2. Condensed Tannins

Condensed tannins possess more than 90% of the global commercial tannin yield [38], which are oligomers and intermediates of flavonoid moieties (Figure 2c). The solubility of condensed tannins in water mainly relies on their flavonoid units. Oligomers consisting of 2–10 flavonoid repetition units can be dissolved in water. However, flavonoid intermediates or polymers with higher molecular masses usually possess low or no solubility in water [13,39]. The flavonoid unit contains two phenolic rings, that is, the A ring and B ring, which possess different chemical reactivity. The A ring involves resorcinol and phloroglucinol moieties, while the B ring contains catechol and pyrogallol moieties [15]. Usually, the nucleophilic centers of the A ring show higher reactivity than that of the B ring, on account of the differences in the position of the Ph-OH groups [11]. The combination of the above two functional rings will endow condensed tannins with various reactivity and properties.

#### 2.2.3. Complex Tannins

Complex tannins (a small branch of the tannins family) are usually composed of ellagitannin and flavonoid moieties [40], which have sophisticated macromolecular structures and limited applications. As a representative example, acutissimin A involves a flavagallonyl moiety joined by a D-glucose derivate via a glucosidic linkage in C_1_ and three ester bonds (Figure 2d).

#### 2.2.4. Phlorotannins

Phlorotannins mainly exist in non-vascular plants (representative examples of phlorotannins are shown in Figure 2e) [32], which can be acquired by the polymerization of phloroglucinol moieties [41], Impressively, their molecular masses range from 126 to 650,000 Da [13]. Based on the type of connection among their monomers, phlorotannins can be broadly divided into four groups, i.e., fuhalols/phlorethols, fucols, fucophloroethols, and eckols (Figure 2a).

### 2.3. Reactivity of Plant Polyphenols

As is well known, the functionalities of plant polyphenols vary from their structures and active groups (including aryl, Ph-OH, carbonyl, carboxylic groups, etc.) [42]. In addition, different types of polyphenol-derived intermediates or chemicals with high performance can also be obtained through reasonable design and chemical modification. Importantly, due to the presence of Ph-OH groups, plant polyphenols can precipitate alkaloids, polysaccharides, and other proteins [43], as well as participating in the polymerization reactions. Additionally, the complex ability of plant polyphenols with metal ions is also receiving increasing attention. Therefore, in this section, the reactivity of plant polyphenols will be discussed in detail.

**Figure 3:**
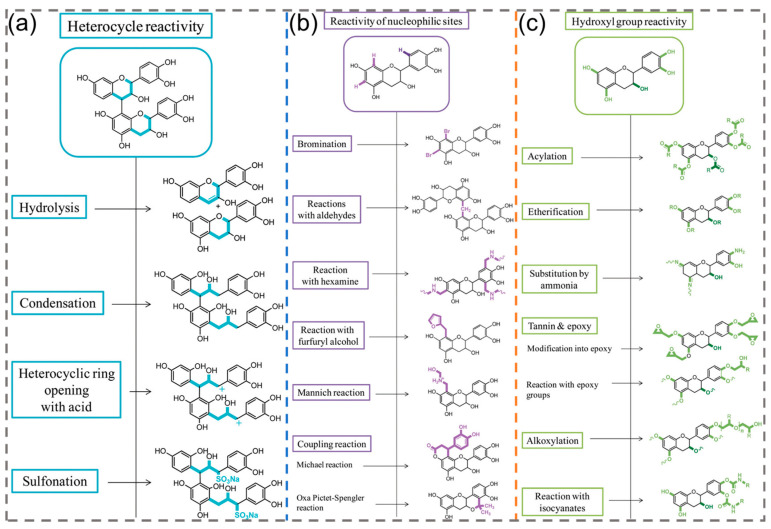
Summary of the chemical reactivity from plant polyphenols (**a**) heterocycles, (**b**) nucleophilic sites, and (**c**) Ph-OH groups. Reprinted from Reference [11] under Creative Commons Attribution 3.0 Unported Licence.

Generally, the heterocycle of plant polyphenols (condensed tannins) can be opened by using different methods (i.e., acidic and alkaline conditions, as well as sulfonation reactions, etc.), which leads to the rearrangements of their chemical structures (Figure 3a) [11]. Specifically, the catechins and anthocyanidins can be obtained through hydrolysis reactions of plant polyphenols under acidic conditions; further, after the hydrolysis stage, the auto-condensation may occur on the basis of nucleophilic reactions. Additionally, alkaline conditions can also be used to induce the rearrangements of plant polyphenols, resulting from the break in the interflavonoid C_4_–C_8_ bond and then re-condensation; further, the sulfonation reaction is feasible for opening the heterocycle of plant polyphenols, which occurs in the existence of hydrosulfate by inserting a sulfonic acid group at a specific position. After incorporating polar groups, the solubility of target plant polyphenols will be improved. Importantly, the sulfonation reaction is beneficial to the extraction of plant polyphenols. In short, these above reactions may further enhance the reactivity of plant polyphenols, which is conducive to the preparation of phenolic derivatives.

The reactivity of nucleophilic sites generated by Ph-OH groups usually leads to an electrophilic substitution (Figure 3b). For example, the bromination reaction of plant polyphenols has been investigated to confirm the reactivity. For (+)-tetra-O-methylcatechin, bromination occurs preferentially at the C_8_ position of the A ring; if the site is occupied, a reaction will take place at the C_6_ position owing to the relatively low reactivity of the B ring. By adjusting the dosage of the brominating agent, substitution can also occur in the C_6_′ position of the B ring. It should be mentioned that the preferential sites depend on the structure and accessibility of flavonoids. In addition, different chemical modifications with aldehydes, hexamine, furfuryl alcohol, etc., can be used to regulate and control the molecular architecture of plant polyphenols. Interestingly, according to the Mannich reaction, amphoteric plant polyphenols can be obtained by reacting with ethanolamines/formaldehyde under acidic media, demonstrating good water solubility [11]. Furthermore, based on the Michael addition reaction, plant polyphenols have shown great potential as structural and functional primitives in creating polyphenol-derived functional intermediates (can be further used to synthesize other chemicals) or materials [44,45].

Ph-OH groups of plant polyphenols also show high reactivity, which can undergo a variety of chemical modifications (including acylation, etherification, substitution by ammonia, etc.) and directly react with epoxy or isocyanate groups (Figure 3c) [46,47,48,49,50,51]. After reaction with these active functions, the chemical reactivity and solubility of plant polyphenols may be further increased. The obtained plant polyphenol derivatives can be regarded as monomers or intermediates to fabricate biobased resins, such as epoxy, polyurethane, polyester, etc. On the other hand, due to the existence of adjacent Ph-OH groups on the benzene ring, plant polyphenols can chelate with metal ions (such as Fe^3+^, Cu^2+^, Al^3+^, etc.) to form stable complexes [52,53], making significant contributions in the fields of plant pigmentation, water purification, nanocoating, and so on. In addition, non-covalent π-π interactions may be formed based on plant polyphenols and other aromatic components (like graphene oxide) [54].

To sum up, based on these above physical or/and chemical reactivities, plant polyphenols have been widely used to construct polyphenol-derived hybrid materials, providing new perspectives and ideas for the field of materials engineering.

## 3. Toward Plant Polyphenol-Derived Materials

By virtue of the high reactivity of plant polyphenols, which can endow the target polymeric materials with favorable physicochemical properties via direct blending, polymerization, coordination, and modification followed by polymerization. Importantly, the unique features of plant polyphenols can also be effectively strengthened or mitigated through material engineering technology [55]. Within this review, some attempts about fabricating plant polyphenol-derived hybrid materials in recent years have been systematically summarized. Particularly, plant polyphenols-metal or/and polymeric materials are discussed in detail, focusing on their chemical architectures, product features, and possible applications in fields such as resistance to fire, water-treatment, functional leather, stimuli-responsive materials, biomaterials, sensing materials, etc.

### 3.1. Materials Based on Polyurethane

As one of the important resins, polyurethanes have been broadly utilized in the fields of antimicrobial, coating, sensing, and flame retardant, etc. [56,57,58]. Nowadays, more and more scientists are paying attention to the design and preparation of biobased multifunctional polyurethanes. As mentioned, the Ph-OH groups of plant polyphenols can react with isocyanate groups via the addition of polymerization to yield biobased polyurethane films, hydrogels, and foams. In this section, the green methods for fabricating plant polyphenol-derived polyurethane materials will be mainly focused on.

#### 3.1.1. Polyurethane Films from Plant Polyphenols

Several studies present the preparation of biobased polyurethane films with unique multifunctionality from plant polyphenols. In the work carried out by Gogoi et al., tannic acid was used as a replacement for fossil-based polyol to fabricate waterborne hyperbranched biobased polyurethane (WHPU) via stepwise polymerization [59]. The thermostabilities and mechanical properties of WHPU films were improved by increasing the tannic acid amounts. Interestingly, benefiting from the hydrolyzable ester bonds of tannic acid, the target WHPU films showed good biodegradability. Similarly, Luo et al. reported a simple method to develop biobased waterborne polyurethane/Ag NP composite films through in situ polymerization, in which tannic acid was used as a reductant and stabilizer to obtain Ag NPs. The results showed that the target polyurethane composite films also possessed improved thermostabilities and mechanical properties. More importantly, the formation of Ag NPs was beneficial for improving their antibacterial performances [60]. Based on the above analysis, plant polyphenols can be directly used to synthesize polyurethanes to improve their thermal/mechanical properties while endowing the resulting polyurethanes with functional properties, such as degradable and antibacterial abilities.

**Figure 4:**
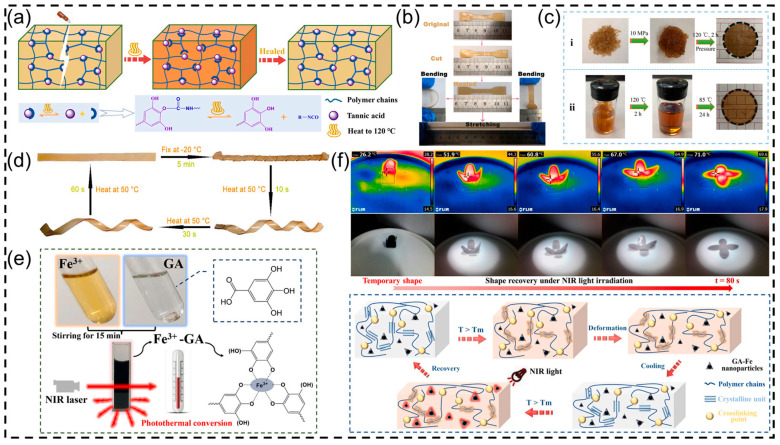
(**a**) Dynamic exchange mechanism of phenol–carbamate bonds, (**b**) self-healing, (**c**) reprocessing (i: hot-pressing molding, ii: solution casting), and (**d**) shape memory abilities of TA-PU. Reprinted from Reference [61] with permission of Elsevier, Copyrights (2021). (**e**) Schematic of the preparation of GA-Fe nanoparticles. (**f**) NIR light induced shape recovery process of the prepared polyurethane nanocomposite film. Reprinted from Reference [64] with permission of Elsevier, Copyrights (2022).

In our previous works [61,62,63], a series of self-healing polyurethane films were designed and prepared, where the thermally reversible phenol–carbamate bonds were directly created based on the polymerization of plant polyphenols (e.g., tannic acid or gallic acid) and isocyanates. As shown in Figure 4a, the dynamic phenol–carbamate bonds will undergo reversible exchange (including dissociation and recombination) at a given temperature, which further endows the tannic acid-based polyurethane (TA-PU) with excellent self-healing and reprocessing abilities (Figure 4b,c). In addition, the phenol–carbamate bonds can also be regarded as “net/crosslinking points” to achieve the shape memory and restoration of the target polyurethane films bearing a reversible phase (soft segment, such as poly-1,4-butyleneadipateglycol (PBA), polyethylene glycol (PEG), polycaprolactone diol (PCL), etc.) (Figure 4d) [61]. Furthermore, our group successfully developed novel iron gallate (GA-Fe) nanoparticles with desired photothermal conversion ability based on the coordination effect between gallic acid (GA) and FeCl_3_ (Figure 4e). Subsequently, GA-Fe nanoparticles were introduced into the GA-based shape memory waterborne polyurethane matrix containing dynamic phenol–carbamate bonds to yield a nanocomposite film, which showed satisfactory near-infrared (NIR) light-triggered shape memory behavior (Figure 4f) and can be effectively reshaped (or reconstructed) by topological rearrangement (rearranging molecular structure) [64]. To sum up, due to the rigid structure and multi-hydroxyl structure of plant polyphenols, the resulting polyurethane films usually showed relatively high crosslinking densities and improved mechanical and thermal properties. Importantly, benefiting from the diversity and reactivity of plant polyphenols, the target polyphenol-based polyurethane films can be endowed with various functions. It can be inferred that with the further development of plant polyphenol-derived polyurethane films (or coatings), they will be applied in more fields.

#### 3.1.2. Polyurethane Hydrogels from Plant Polyphenols

**Figure 5:**
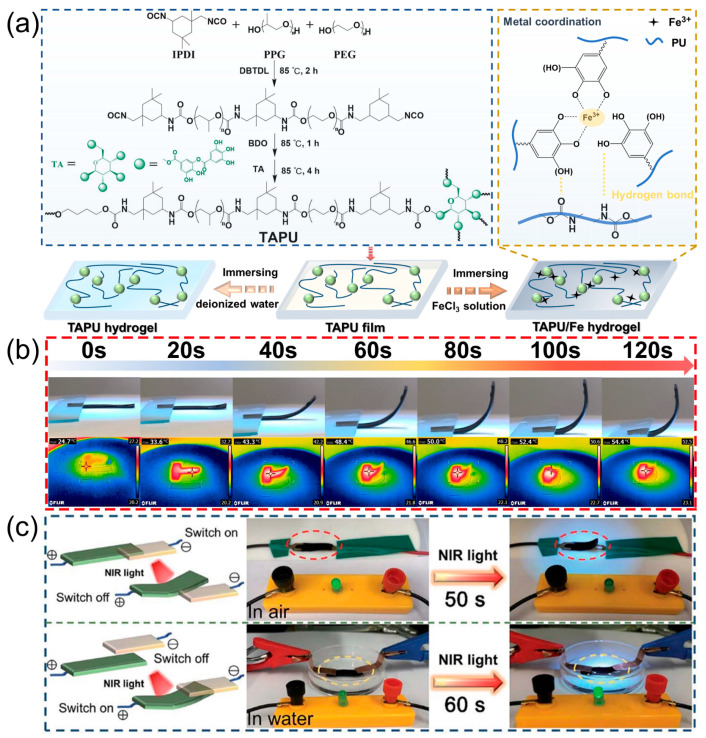
(**a**) Schematic of the preparation procedure, (**b**) NIR light-responsive bending behavior, and (**c**) application (worked as light-driven switch) of the target polyurethane hydrogel. Reprinted from Reference [69] with permission of Royal Society of Chemistry, Copyrights (2022).

Polyurethane hydrogels with network structures are widely used in our daily life due to their good mechanical strength, ideal toughness, and non-toxicity [65], which can be fabricated by incorporating PEG into the polyurethane framework serving as the hydrophilic component [66]. Interestingly, plant polyphenols can be used as crosslinking agents to create network structures in polyurethane hydrogel skeletons for replacing fossil-based monomers. For example, Divakaran and co-workers prepared a series of dual-crosslinked polyurethane hydrogels, in which 1,2,6-hexanetriol (HT) and curcumin were used as crosslinking agents. The results exhibited that their swelling degree and mechanical properties can be adjusted by varying the content of HT and curcumin. In addition, the target hydrogel had good biocompatibility and antibacterial properties, manifesting great application potential in the field of tissue engineering [67]. Similarly, Wen et al. reported a novel fluorescent polyurethane hydrogel by using PEG, isophorone diisocyanate (IPDI), polyphenol compound resveratrol (3, 4, 5-trihydroxystilbene), and trimethylolpropane, which showed the desired swelling ratio (607%) and high mechanical properties (tensile strength of 1.87 MPa, breaking elongation of 801%, and toughness of 8.33 KJ/m^2^) [68]. To endow the resulting plant polyphenol-derived polyurethane hydrogel with special functional properties, some extra elements can be incorporated into the matrix. In the early work, we reported a novel stretchable, strain sensitive, and NIR-responsive polyurethane hydrogel based on the phenol–carbamate network and Fe^3+^-polyphenol coordinative network (Figure 5a). The target hydrogel can be worked as an NIR light-triggered actuator, due to the existence of the thermosensitive PEG-PPG (polypropylene glycol) blocks and Fe^3+^-polyphenol component with photothermal conversion ability (Figure 5b). Importantly, after introducing Fe^3+^ ions, the prepared hydrogel showed improved ionic conductivity and can be designed as a light-driven switch (Figure 5c) [69]. Inspired by the construction of the Fe^3+^-polyphenol coordinative network, plant polyphenol-based polyurethane hydrogels have the potential to be used for adsorbing heavy metal ions in sewage, due to the excellent chelating ability of plant polyphenols with various metal ions. To sum up, the above findings indicate that it is feasible to prepare environment-friendly plant polyphenol-derived polyurethane hydrogels, which provides an ecological alternative way to meet the performance requirements of modern materials. By combining biological plant polyphenols with other functional elements, the final biobased polyurethane hydrogels will undoubtedly show broader application prospects.

#### 3.1.3. Polyurethane Foams from Plant Polyphenols

**Figure 6:**
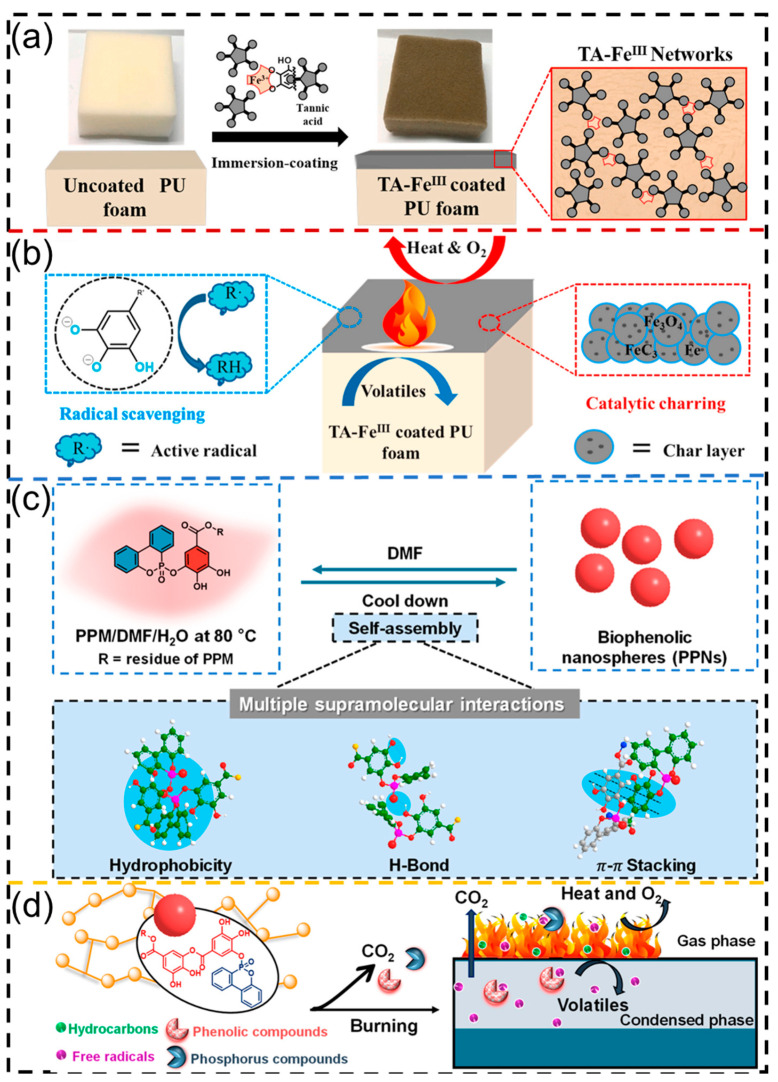
(**a**) Schematic of the preparation of tannic acid-Fe^3+^ network coated polyurethane foam. (**b**) Schematic of flame retardant mechanism of tannic acid-Fe^3+^ network. Reprinted from Reference [74] with permission of Elsevier, Copyrights (2021). (**c**) Schematic of the synthesis of biophenolic nanospheres via multiple supramolecular interactions. (**d**) Schematic of the flame retardant process of biophenolic nanospheres. Reprinted from Reference [75] with permission of American Chemical Society, Copyrights (2023).

Biobased polyurethane foams are an environmentally friendly alternative to traditional commercial foams, owing to their good bacteriostatic activity, biodegradability, and relatively low price [70,71]. Plant polyphenol-based polyurethane foams usually consist of various plant polyphenols (or their derivates) and isocyanates. It should be mentioned that the derivatization of plant polyphenols has been attempted to make the Ph-OH groups more accessible prior to reacting with isocyanates. A novel polyurethane foam was synthesized starting from wattle tannin after being liquefied and methylene diphenyl diisocyanate (MDI), which showed desired biodegradation and bacteriostasis [72]. Maria Oliviero and co-workers used condensed tannins with different concentrations as the polyol segments to yield polyurethane foams; they found that the ability of target products to resist UV radiation was enhanced with an increase in the tannin amounts, due to the aromatic compound bearing unsaturated bonds that can effectively absorb UV radiation [73]. Unfortunately, the mechanical properties decreased with the addition of the tannin amounts. On the other hand, plant polyphenols can also be introduced into polyurethane foams as functional components via simple adsorption or the blending method. For example, Yang et al. first synthesized a green flame retardant based on the tannic acid-Fe^3+^ metal–phenolic network and then loaded on the surface of polyurethane foams (Figure 6a). Owing to the good carbonization capacity and ideal free radical scavenging ability of tannic acid, as well as the catalytic effect of Fe^3+^ ions on the charring process (Figure 6b), the prepared polyurethane foams exhibited excellent flame retardancy performance [74]. In addition, Wang et al. reported multifunctional biophenolic nanospheres based on the temperature-controlled self-assembly between 9,10-dihydro-9-oxa-10-phosphaphenanthrene 10-oxide (DOPO) and tannic acid (Figure 6c), which can be homogeneously incorporated into the framework of polyurethane foams via a simple blending method during foaming. The obtained nanocomposites showed desired aging resistance, mechanical reinforcement, and flame resistance (Figure 6d). Importantly, the biophenolic nanospheres can be effectively decomposed into their precursors under certain conditions and recycled from the matrix [75], which affords a new sustainable strategy for constructing multifunctional flame-retardant nanocomposites. Meanwhile, we believe that the utilization of cellulose/lignin (bearing hydroxyl groups) as a biological source in the preparation of sustainable polyurethane foams can also provide a reference for the design of plant polyphenol-based multifunctional polyurethane foams.

Impressively, the above polyurethane foams derived from plant polyphenols provide an ecological alternative for creating semi-biobased systems, but the use of isocyanates still has significant disadvantages (especially for toxicity). In recent years, non-isocyanate polyurethane has experienced rapid development due to the isocyanate monomers; e.g., MDI and toluene diisocyanate (TDI) are classified as toxic and carcinogenic chemicals, recognized by the European Community [76]. M. Thébault and co-workers reported a kind of biobased non-isocyanate foam, in which the hydrolysable chestnut tannin bearing gallic acid unit was first combined with dimethyl carbonate monomer and then further reacted with hexamethylenediamine. The ^13^C NMR and FT-IR spectra showed the existence of a urethane unit in the target product [77]. Similarly, in work carried out by Chen et al., mimosa tannin was used as a natural fire retardant to enhance the fire resistance of glucose-based non-isocyanate foam by partially replacing glucose. The FT-IR analysis exhibited that the urethane linkage was successfully constructed and the chemical architecture of the condensed tannin-glucose-based non-isocyanate foam was still preserved, even if reducing the glucose amounts [78]. Therefore, using the above natural polyphenol materials to construct biobased polyurethane foams can effectively reduce the dependence on toxic isocyanates.

In short, the comprehensive properties of the obtained plant polyphenol-derived polyurethane materials (including films/coatings, hydrogels, and foams) can be adjusted by selecting different polyphenol structures, changing the polyphenol proportion, and introducing other functional components. Green chemistry approaches to achieve the manufacture of biobased polyurethane materials may be the future development trend, especially for non-isocyanate polyurethane materials, as their synthesis process is relatively green, and they have similar or better mechanical and thermal properties compared to traditional polyurethanes. However, due to the relatively weaker reactivity of carbonate groups compared to isocyanate groups [79], the physical or/and chemical properties of plant polyphenol-derived non-isocyanate polyurethanes may be more difficult to regulate than those of diisocyanate-based polyurethanes. Therefore, more refined intermediates or chemicals derived from plant polyphenol-based biorefineries deserve further attention, so as to create multifunctional biobased polyurethane materials with high performance for meeting diverse requirements.

### 3.2. Materials Based on Epoxy Resin

As one of the most representative thermosetting polymers, epoxy resins possess the desired mechanical and thermal properties, good chemical resistance, as well as ideal processability [80,81,82], on the basis of an adjustable internal crosslinking network formed of different kinds of epoxy monomers and curing agents [83] Consequently, epoxy resins have been widely used in various fields, such as coatings, adhesives, constructions, etc. [84,85,86,87,88]. In recent years, biobased epoxy resins have received increasing attention from researchers. Interestingly, renewable and sustainable plant polyphenols can be used to replace the toxic monomer (such as bisphenol A) in the synthesis of epoxy resins. For instance, Benyahya et al. proposed a new method for the synthesis of biobased epoxy polymers based on green tea polyphenols. Firstly, the green tea polyphenols were modified by epichlorohydrin to yield the epoxy pre-polymers (GEGTE). Subsequently, the GEGTE pre-polymers were combined with isophorone diamine (IPD) to create the polyphenol-derived epoxy resin (GEGTE-IPD), which possessed higher crosslinking density and thermal stability. In addition, compared with fossil-based epoxy polymers composed of bisphenol A and IPD, GEGTE-IPD exhibited a higher glass transition temperature and better mechanical properties [89]. Similarly, Long et al. fabricated a polyphenol-based epoxy resin monomer (pyrogallol triglycidyl ether) from biological pyrogallol by combining epichlorohydrin with olefin epoxidation, which can be further cured using methyltetrahydrophthalic anhydride. Importantly, the target epoxy resin shows ideal thermomechanical properties (i.e., glass transition temperature of 164 °C, storage modulus of 3500 MPa) [90]. The above-mentioned studies demonstrate that the addition of plant polyphenols can effectively improve the thermal and mechanical properties of the resulting epoxy resins, providing new methods for creating polyphenol-derived epoxy resins. Meanwhile, the green preparation concept of epoxy resins will also receive increasing attention.

**Figure 7:**
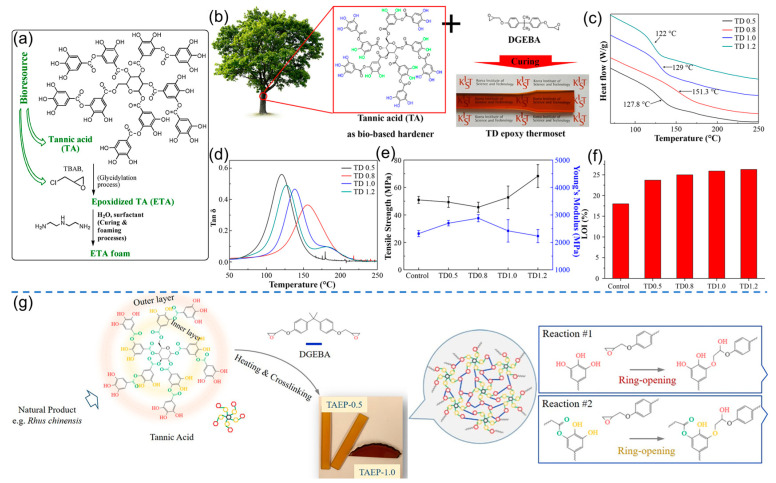
(**a**) Preparation of tannic acid-based thermosetting epoxy (ETA) foam. Reprinted from Reference [91] with permission of Elsevier, Copyrights (2018). (**b**) Synthesis of thermosetting epoxy resin from tannic acid and DGEBA. (**c**) DSC curves, (**d**) tan δ, (**e**) mechanical properties, and (**f**) LOI values of thermosetting epoxy resin with different amounts of tannic acid. Reprinted from Reference [92] with permission of American Chemical Society, Copyrights (2019). (**g**) Schematic of the reaction between tannic acid and DGEBA. Reprinted from Reference [93] with permission of American Chemical Society, Copyrights (2020).

In addition, plant polyphenols can also be used as biobased flame retardants and hardeners to prepare high-performance epoxy resins. In work reported by Esmaelli et al., a novel epoxidized tannic acid (ETA) foam was designed and fabricated. Specifically, tannic acid was first epoxidized via glycidylation and then participated in the subsequent foaming and curing processes to obtain the target ETA foam with a yield of 98% (Figure 7a). The ETA foam showed relatively lower thermal conductivity (0.0286 W m^−1^ K^−1^), enhanced char yield (48.3% in air), and a higher limiting oxygen index (LOI) value (36.8%), which can be regarded as a self-extinguishing material [91]. Similarly, Kim et al. fabricated a tannic acid-based thermosetting epoxy resin, in which tannic acid served as both a flame retardant and a renewable hardener (Figure 7b). In this work, the target biobased epoxy resin exhibited the desired thermal and mechanical properties (i.e., glass transition temperature of 155.4 °C, Young’s modulus of 2900 MPa), as displayed in Figure 7c–e. In addition, with an increase in tannic acid amounts, the resulting resins showed improved flame-retardant properties (Figure 7f), as the tannic acid can act as an oxygen free radical quenching agent and a carbonization agent [92]. In addition, Feng et al. reported the synthesis of a biobased thermosetting epoxy resin, in which tannic acid with diverse bonding capacities and unique branched structure can be regarded as a multifunctional curing agent (Figure 7g). The results demonstrated that it is possible to prepare epoxy resin with high mechanical properties (tensile strength of 98.4 MPa) and tunable functionalities, including damping, recyclability (based on the transesterification process), and shape memory (based on the entropic elasticity) [93]. The above design concepts may provide new and promising insights for the fabrication of biobased high-performance multifunctional epoxy polymers.

In summary, different kinds of plant polyphenols have been employed to construct environmentally friendly epoxy monomers and resins. However, it should be mentioned that some plant polyphenols derived from different resources usually need to be modified or functionalized based on their active Ph-OH groups for further curation. Limited by the varied reactivity and complicated structure of plant polyphenols, the synthesis processes of biobased epoxy resins may not be easily reproducible in industry and difficult to apply in fine chemicals. To this end, some attempts are being made to depolymerize complex plant polyphenols and use relatively low-molecular-weight monomers for creating epoxy derivatives. Based on these green synthetic techniques, more and more fully biobased epoxy resins from plant polyphenols will emerge and be applied in different fields.

### 3.3. Materials Based on Phenol-Aldehyde Polymer

The combination between phenols and aldehydes has been broadly explored at an industrial level to achieve the preparation of coatings, adhesives, foams, and composites [94,95]. In the synthesis of phenol-aldehyde polymers, biobased plant polyphenols can be used to improve the physicochemical properties of the resins. For example, due to the good affinity of plant polyphenols to aldehydes, the obtained phenol-aldehyde adhesives can show high weather resistance and boiling resistance, as well as a low formaldehyde release rate [35]. In recent years, hydrolyzed tannins and condensed tannins have been widely explored in the field of adhesives. However, it is worth mentioning that the reactivity of hydrolyzed tannins with aldehydes is relatively low, and their connection units (such as ester bonds) possess relatively lower resistance to moisture [38]. In contrast, condensed tannins show better resistance to moisture owing to their units linked by C-C bonds, which are widely used in the synthesis of adhesives and foams. In work carried out by Hafiz et al., the preparation of phenol-formaldehyde adhesives with different tannin (from Acacia mearnsii bark) concentrations was reported. The results showed that the introduction of tannin into the phenol-formaldehyde prepolymer accelerated the gelation time and increased the viscosity of the resins. Importantly, the dynamic thermomechanical analysis (DMA) results showed that the stiffness of all the target resins improved with the addition of tannin [96]. In another study carried out by F. Santiago-Medina et al., the preparation of phenol-aldehyde adhesives for wood panels based on the reaction of procyanidin-type condensed tannins (from pine bark) and renewable vanillin-derived dialdehydes was reported. In this work, the target adhesive showed ideal gelation time, and it was suitable for fabricating particleboards (167 s at pH 10). This study provides a novel method to create totally non-toxic and environmentally friendly resins on the basis of renewable plant polyphenols and their derivatives [97]. The reaction between condensed tannins and aldehydes can be catalyzed by an acid or base. Usually, the A ring (nucleophilic centers) is more reactive than the B ring, and the reaction of the B ring with aldehydes can be activated under alkaline conditions (approximately pH 10). It should be mentioned that the fast reaction of the A ring with aldehydes makes the subsequent process difficult to control (such as molecular weight, structure, viscosity, etc.); thus, the content of plant polyphenols in the system is limited to a low level for inhibiting the condensation reaction. Due to the incomplete polymerization, rigid and brittle resins (especially for phenol-aldehyde adhesives with unsatisfactory adhesion performance) may be obtained. To this end, the depolymerization of plant polyphenols before being used to synthesize phenol-aldehyde resins can effectively improve the performance of the target product. For instance, Li et al. reported a novel biobased adhesive (phenolic resin) with high performance, in which Acacia mangium tannin was depolymerized and then reacted with polyethyleneimine. The target adhesive exhibited shorter gelation time (67.6%) and fewer formaldehyde emissions (64.4%), as well as enhanced bonding strength (63.6%), compared with the control adhesive, owing to the ideal chemical reactivity of depolymerized tannin [95]. Hopefully, the as-prepared biobased adhesive may be used as a substitute for traditional phenolic resins.

It is also possible to prepare phenol-aldehyde foams based on plant polyphenols. The traditional plant polyphenol-derived foam formulation mainly includes the following ingredients: plant polyphenol extract, formaldehyde, furfuryl alcohol (serving as an exothermic agent), blowing agent, water, additives, etc. In addition, glyoxal (a non-toxic di-aldehyde) can be regarded as a formaldehyde substituent to synthesize phenolic foams, avoiding the release of harmful gases. In work reported by Zhou et al., pine tannin was used as a biobased component, and glyoxal was used to completely replace formaldehyde for synthesizing eco-friendly pine tannin/furanic rigid foams, which exhibited low thermal conductivity and desired mechanical properties [98]. As we know, depending on the formulation, plant polyphenol-derived foams can be deigned as rigid or semi-rigid types. It has been reported that the size and structure of the pores can be controlled by regulating the content of plant polyphenols (tannins) [99].

To sum up, all kinds of works carried out to date about biobased phenol-aldehyde resins/foams composed of plant polyphenols are very promising, and the obtained products also show comparable thermal or/and mechanical properties to those current petroleum-based phenol-formaldehyde resins/foams.

### 3.4. Materials Based on Polyester

**Figure 8:**
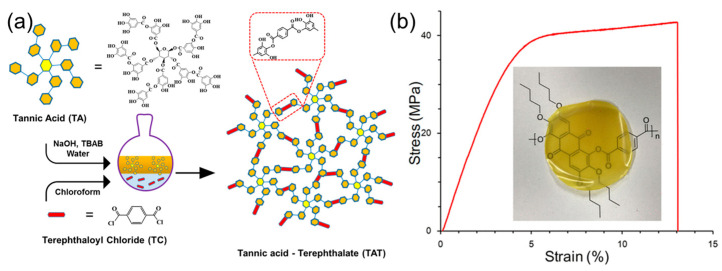
(**a**) Schematic of the preparation of tannic acid-terephthalate. Reprinted from Reference [101] with permission of Elsevier, Copyrights (2018). (**b**) Stress–strain curve of the representative biobased polyester from gallic acid. Reprinted from Reference [103] with permission of American Chemical Society, Copyrights (2019).

The preparation of plant polyphenol-derived polyesters is feasible via the reaction between plant polyphenols and different types of acylating agents such as carboxylic acids, acid anhydrides, and highly reactive derived monomers (e.g., acid halides) [100]. The carbonization ability of plant polyphenols has been used in the preparation of polyesters with flame retardancy. In work carried out by Xia et al., the synthesis of flame-resistant bio-derived compounds from tannic acid was reported. As shown in Figure 8a, tannic acid underwent a crosslinking reaction via interfacial polymerization with terephthaloyl chloride to obtain tannic acid-terephthalate, which exhibited good thermal stability (less than 3% mass loss at 230 °C), higher char yield of 36.4%, and low heat release ability (<80 J g^−1^ K^−1^). After coating tannic acid-terephthalate on nylon 66, the fabric showed rapid self-extinguishing capacity and a decreased char length in vertical combustion tests, due to the heat-resistant carbon layer created by tannic acid-terephthalate, which can effectively inhibit the flame propagation [101]. This work confirms that tannic acid-terephthalate can be used as an ideal flame retardant to improve the fire resistance of fabric.

Plant polyphenols can also endow polyesters with good biodegradability. For example, P. Song and co-workers developed a novel biodegradable polyester on the basis of tannin-grafted polycaprolactone (TA-g-PCL). The results showed that the thermodynamic properties and dissolubility of TA-g-PCL polymer varied obviously with its molecular weight. With the growth of PCL monomer segments, the TA-g-PCL polymer was converted from the amorphous to crystalline state. At the same time, the dissolubility of the target TA-g-PCL polymer in chloroform was also significantly improved, which provided a possibility for its further application in the biomedical field [102]. Altogether, this work presents a new perspective on the synthesis of novel biodegradable polyesters derived from plant polyphenols. Biobased polyesters can also be obtained through an interfacial polymerization reaction between gallic acid and five dicarboxylic dichlorides. The solubility and thermal properties of the prepared polyesters can be flexibly adjusted by selecting appropriate alkoxy groups and aromatic dicarboxylate units. Through special structure design, the glass transition temperature of the prepared polyesters can be controlled between 81 °C and 308 °C. In addition, as shown in Figure 8b, the representative polyester exhibited the desired mechanical properties (elongation of 13.1% and Young’s modulus of 1.1 GPa) [103]. In another study, Hakkarainen et al. developed sustainable polyesters based on a eugenol-modified 1,3-Dioxolan-4-one component. The target polyester showed the desired thermal stability (210 °C) and improved tensile strength (70.8 MPa), owing to the presence of the aromatic-rich structure in the system. In addition, the obtained polyester can be degraded in alkaline water. Importantly, benefiting from the transesterification between free hydroxyl groups and carboxyl groups, the prepared polyester showed ideal self-healing and shape memory abilities [104].

Altogether, plant polyphenol-based polyesters can be simply fabricated by the reaction of plant polyphenols (and their derivatives) with different monomers (e.g., acid anhydrides, acid halides, fatty acids, etc.), which have been proven to show good flame retardancy, biodegradability, UV resistance, and so on. However, some functional polyesters obtained from plant polyphenols possessed relatively low flexural properties. Thus, for the next generation of plant polyphenol-derived polyesters, more attention should be paid to the balance of comprehensive performance.

### 3.5. Materials Based on Leather

**Figure 9:**
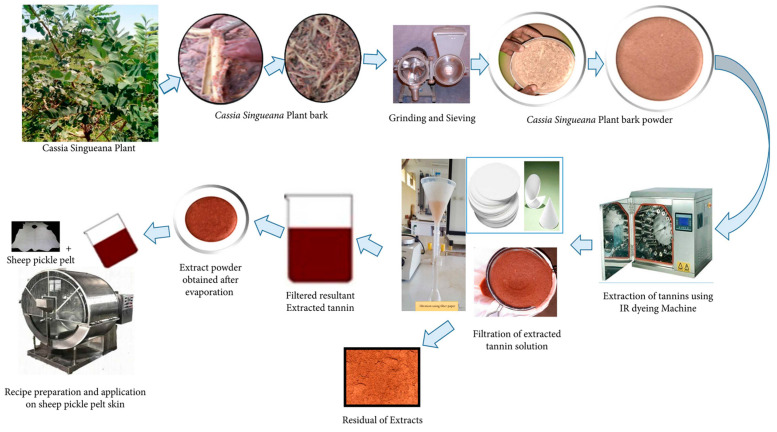
Extraction of vegetable tannins from Cassia singueana bark. Reprinted from Reference [105] under Creative Commons License 4.0.

**Figure 10:**
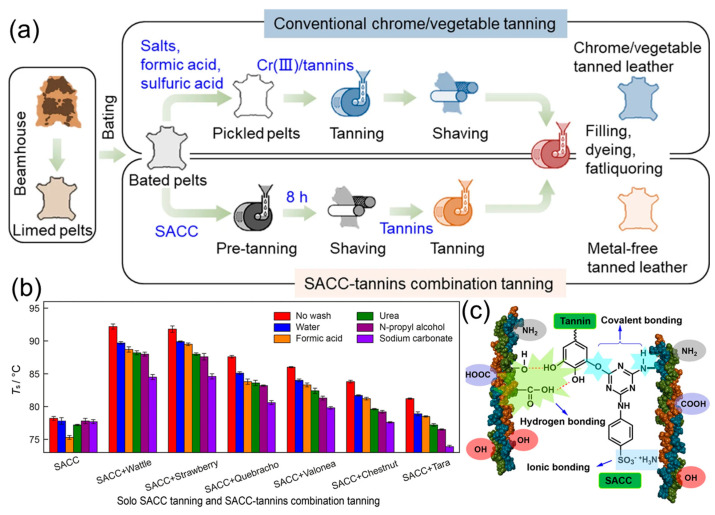
(**a**) Schematic of traditional chrome/vegetable tanning procedure and SACC-plant polyphenols combination tannage. (**b**) Shrinkage temperature (T_s_) of the obtained leather after being washed with different media. (**c**) Schematic of the interactions of SACC and plant polyphenols with collagen. Reprinted from Reference [106] under Creative Commons License 4.0.

As mentioned before, plant polyphenols can be regarded as vegetable tanning agents to achieve the conversion of hide into leather. In recent years, the interaction between plant polyphenols and collagens has been one of the research hotspots in the field of leather chemistry. In work reported by Teklemedhin et al., vegetable tannin was first extracted from Cassia singueana bark through a simple water extraction method (Figure 9) and then further used for sheep pickle pelt. The obtained leather tanned by Cassia singueana tannin exhibited comparable physicochemical properties to traditional Mimosa tanned leather. For example, the shrinkage temperature, tensile strength, and elongation at break of the resulting leather reached up to 83 °C, 15.6 N/mm^2^, and 45.3%, respectively, which were slightly superior to those of the control sample (tanned leather with Mimosa extract) [105]. These results demonstrated that Cassia singueana extract can be used as an alternative eco-friendly tannin to alleviate the pollution caused by chrome tanning in the leather industry. Further, in work reported by Xiao et al., a sustainable metal-free combination tannage method based on triazine-based syntan bearing chlorine groups (SACC) and plant polyphenols (i.e., wattle, tara, quebracho, etc.) was developed (Figure 10a). The obtained leather exhibited a high shrinkage temperature (~92 °C) that did not change obviously, even after being washed with water, urea, and n-propanol solution (Figure 10b). It may be due to the fact that the SACC and plant polyphenol (wattle) can effectively diffuse into collagen fibers and bind to the collagen through covalent bonds, hydrogen bonds, and ionic bonds (Figure 10c) [106]. This work not only provides new ideas for improving the organoleptic and physicochemical properties of chrome-free leather products but also reduces the influences of chromium wastes to the environment and human health. In addition, in our previous work, a simple chrome-free combination tanning method by using silicic acid and plant polyphenols was proposed. The combination-tanned leather exhibited good thermal stability, desired mechanical properties and softness, as well as ideal storage stability compared to the control leather tanned by silicic acid alone. During the storage process, the Ph-OH groups of plant polyphenols can form a hydrogen bonding interaction with Si-OH groups to inhibit their excessive condensation. Importantly, the environmental impact assessment (including BOD_5_/COD, TS, DS, and SS) of tanning wastewater demonstrated that this combination-tanning method is a green and sustainable tanning technique [107]. With increasingly strict environmental regulations and policies, as well as the strengthening of people’s health awareness, the green tanning technology based on plant polyphenols will be further developed and applied in the leather field.

Plant polyphenols can not only be used for leather tanning but also used as functional components to endow leather products with the desired properties. In work reported by Marsal et al., the effect of plant polyphenols (mimosa, quebracho and tara) in the decrease of the formaldehyde concentration in leathers tanned by resin (melamine-formaldehyde or dicyandiamide-formaldehyde) was investigated. The results showed that mimosa possessed the desired capacity to decrease the formaldehyde concentration of the resin-tanned leathers, and the ability was enhanced with ageing [108]. This may be due to the high reactivity of plant polyphenols towards formaldehyde. Furthermore, Yu et al. fabricated a novel high-performance water purification membrane based on tannic acid and collagen fibers derived from animal hides, in which tannic acid was used as a vegetable tanning agent to functionalize collagen fibrils inspired by the traditional leather tanning technique. The resulting composite showed ideal antibacterial properties because of the generation of reactive oxygen species (ROS) based on the catechol unit and possessed excellent water disinfection efficiency (>99.9%, ∼150 L m^−2^ h^−1^) [109], which may provide new ideas for the preparation of green and antibacterial microfiltration membranes. In addition, Yan et al. reported a novel high-performance X-ray shielding material based on a microfiber membrane (a kind of synthetic leather with hierarchical structure) via the impregnation–desolvation strategy and coating process. In this work, with the assistance of plant polyphenols, the rare earth (Ce or Er) element can be effectively loaded and uniformly dispersed into the microfiber membrane. The obtained composites showed an average 10% X-ray attenuation efficiency higher than the control sample excluding polyphenols, and an improvement of 9% in X-ray attenuation efficiency compared to that without hierarchical structure. Due to the synergistic effect between plant polyphenols and the hierarchical structure, the target composite bearing uniformly dispersed rare earth element showed an average improvement of 19% in X-ray attenuation efficiency compared to that of lead sheet [110]. Inspired by this work, leather/collagen-based products with X-ray shielding capability have the potential for further application in the clinical field.

To sum up, as a rich and renewable resource, plant polyphenols can be used as vegetable tanning agents to reduce the contamination caused by chrome tanning agents and can also be worked as functional components to endow leather with satisfactory properties, showing great potential for application in the leather industry.

### 3.6. Materials Based on Hydrogel

As a kind of soft material, hydrogels composed of a crosslinked network and hydrophilic component can absorb and retain an adequate amount of water [111,112,113]. Due to the unique properties (such as good biocompatibility, ideal water absorption, and tunable mechanical properties), hydrogels have been widely applied in various fields, including medicine, tissue engineering, sensors, and actuators [114,115,116,117,118,119]. In recent years, there have been more and more reports on the preparation of hydrogels from plant polyphenols. Benefiting from their excellent properties, the obtained plant polyphenol-derived hydrogels will also show various functions.

**Figure 11:**
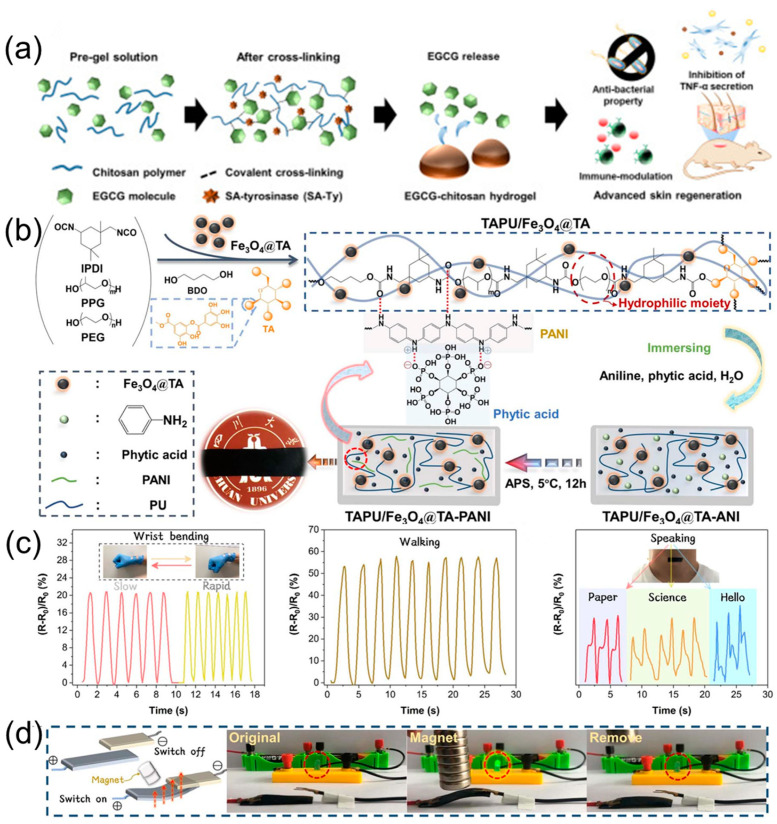
(**a**) Schematic of the preparation of EGCG-chitosan hydrogel. Reprinted from Reference [121] with permission of Elsevier, Copyrights (2020). (**b**) Schematic of the preparation of nanocomposite hydrogel. (**c**) Relative resistance changes of the target organohydrogel for monitoring human movements. (**d**) Photography of an electrical switch triggered by using a magnet. Reprinted from Reference [126] with permission of Royal Society of Chemistry, Copyrights (2023).

For instance, in work reported by Zhang et al., a robust and fully green hydrogel with a water content of 54% was successfully prepared based on chitosan and tea polyphenols, which showed good antibacterial and antioxidant properties as well as UV resistance and can be applied to soft contact lens [120]. Fully green polyphenol-derived hydrogels may show desired biocompatibility and have important research value in the biomedical field. Furthermore, plant polyphenols can induce immunity in humans and inhibit external infections. Kim et al. developed a novel biobased hydrogel containing epigallocatechin gallate (EGCG) and chitosan through a one-pot method (Figure 11a); its crosslinked degree and physical properties can be adjusted by the EGCG concentrations. Importantly, the obtained hydrogel showed desired antibacterial and antioxidant properties, which can be utilized in full-skin defect wounds to promote skin regeneration due to the EGCG having the ability to scavenge free radicals and suppress inflammation [121]. Moreover, in work carried out by Deng et al., an agarose-based nanocomposite hydrogel with tannic acid-Fe^3+^ component was developed, which showed excellent mechanical properties and biocompatibility, as well as an ideal photothermal effect (58 °C, irradiating for 10 min under NIR light). Interestingly, the prepared hydrogel can kill ~99% of bacteria after being irradiated by NIR light for 10 min and effectively promote wound healing [122], showing great potential as a functional wound dressing in the biomedical field. The above studies demonstrated that the introduction of plant polyphenols can regulate the intrinsic properties of the target hydrogels, and the further construction of a coordinative network based on plant polyphenols and metal ions will also endow hydrogels with special functionality.

Recently, some other multifunctional hydrogels (such as self-adhesives, sensors, and actuators) have also been designed based on plant polyphenols. For example, Fan et al. prepared a self-adhesive, antibacterial, and recyclable dual-network hydrogel based on PVA/chitosan/cyclodextrin/black wattle tannin (a kind of low-cost plant polyphenol), which exhibited ideal mechanical properties and fatigue resistance, as well as high sensitivity (GF = 4.87). Importantly, the incorporation of black wattle tannin into the hydrogel matrix can endow it with excellent and repeated adhesion. The resulting hydrogel can be worked as a flexible strain sensor to detect human movements [123]. Similarly, in work reported by Liu et al., a dual-network interpenetrating conductive hydrogel based on poly(acrylic acid), methacrylic acid-functionalized chitosan, Fe^3+^ ions, and tara tannin was successfully synthesized, which possessed high transmittance, ideal UV absorption capacity, good mechanical properties (900%, 0.75 MPa), and desired adhesion. In addition, its excellent self-healing ability and strain sensitivity (GF = 5.33) made it possible to serve as a conductive strain sensor [124]. Zhang et al. fabricated an NIR-responsive composite hydrogel by introducing Fe^3+^/tannic acid (photothermal agent) into the poly(N-isopropylacrylamide) matrix. The obtained hydrogel exhibited good mechanical properties and photothermal effects due to the formation of a metal–phenolic network based on Fe^3+^ ions and tannic acid. Benefiting from the desired thermosensitivity (the volume phase transition temperature is about 32 °C) and photothermal conversion ability, the resulting hydrogel can achieve the deformation behaviors under NIR irradiation and can be used as NIR-responsive actuators [125]. Combining the advantages of plant polyphenols with other functional components will undoubtedly further expand the application area of polyphenol-based hydrogels. In our previous work, a magnetic-responsive, environmentally stable, and conductive organohydrogel was successfully prepared. Firstly, Fe_3_O_4_ nanoparticles were modified by tannic acid and then further introduced into a tannic acid-based polyurethane matrix bearing PEG units (hydrophilic component) through in situ polymerization. Subsequently, the as-prepared nanocomposite film was soaked in an aqueous solution containing aniline and phytic acid to obtain the nanocomposite hydrogel with improved conductivity (Figure 11b). Finally, the target nanocomposite hydrogel was converted into organohydrogel through a solvent exchange strategy, which showed ideal conductivity (0.36 mS cm^−1^), mechanical properties (371%, 0.83 MPa), environmental tolerance, and electromagnetic shielding effectiveness. Interestingly, the resulting organohydrogel can be worked as a strain sensor to monitor human movements (Figure 11c) and designed as a magnetically controlled switch to achieve circuit connection (Figure 11d) [126].

To sum up, plant polyphenols can be broadly used for preparing multifunctional hydrogels (e.g., as wound dressing, strain sensors, and stimuli-responsive actuators) due to their unique features, including biocompatibility, high reactivity, adhesion, chelating ability, and so on. We believe that with the deepening of research on plant polyphenol–material system, more and more intelligent hydrogels will appear and show great application potential in various fields.

### 3.7. Materials Based on Nanoparticles

**Figure 12:**
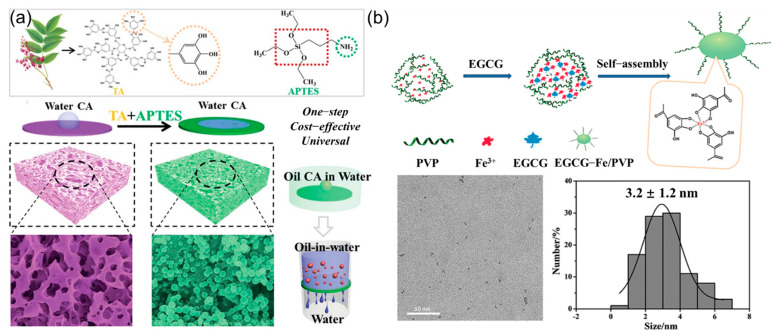
(**a**) Schematic of the “hydrophobic-to-superhydrophilic” change of commercial films based on one-step co-deposition strategy. Reprinted from Reference [128] with permission of Royal Society of Chemistry, Copyrights (2018). (**b**) Schematic of the synthesis of EFPP nanoparticles with ultra-small size. Reprinted from Reference [129] with permission of Royal Society of Chemistry, Copyrights (2019).

Nowadays, organic/inorganic/organic–inorganic nanoparticles are widely applied in the catalysis, electronics, environment, medicine, and composite material fields. Plant polyphenols can also be used to prepare nanoparticles based on their self-polymerization or complexation reaction with metal ions. In work reported by Zhao et al., novel poly(tannic acid) nanoparticles with intrinsic fluorescence were successfully prepared based on the self-polymerization of tannic acid, which exhibited good water dispersibility, ideal biocompatibility, and biodegradability. Importantly, the fluorescence of the obtained poly(tannic acid) nanoparticles can be quenched using picric acid, demonstrating a sensitive and rapid detection ability (as a fluorescent sensor) [127]. In addition, Yang and co-workers fabricated a series of natural polyphenol-based fluorescent polymer dots through a one-pot co-polymerization strategy without an external energy input, which showed ideal photostability and biocompatibility. The luminescence performances of the target polymer dots varied with the structure of polyphenols. Importantly, the resulting polymer dots possessed high selectivity towards Cu^2+^, which can be applied in the detection of Cu^2+^ (as fluorescent probes) in living organisms and environmental water samples [128]. This work provides a new idea and sustainable avenue for the creation of biomass fluorescent polymer dots derived from plant polyphenols, which shows great potential in the biomedical and environmental applications (especially for pollutant detection). In another work, a novel template-mediated supramolecular assembly strategy was proposed to prepare protein-polyphenol-based nanoparticles [129]. After removing the mesoporous template, the obtained biomass nanoparticles can achieve charge reversal (in acidic condition) and glutathione-induced disassembly via competitive supramolecular interactions, which can be used for intracellular protein delivery. In the field of biomedicine, the intracellular delivery of proteins has been regarded as a promising strategy to control cellular behavior. Currently, although some intracellular delivery methods have been developed, designing a polyphenol-based versatile delivery system that can respond to different physiological triggers through simple chemical synthesis should be given more attention. In another interesting work reported by Wang et al., a facile one-step strategy was proposed to achieve the “hydrophobic-to-superhydrophilic” change of commercial films through the co-deposition of 3-aminopropyltriethoxysilane (APTES) and tannic acid. Benefiting from the good adhesion of tannic acid, the hydrophilic and hierarchical nanospheres formed by self-assembly (based on Michael addition reaction and Schiff’s base reaction) can be loaded onto the hydrophobic films (such as PVDF, PTFE, and PP). Moreover, the obtained superhydrophilic commercial films achieved the high-efficiency separation of oil-in-water emulsions (Figure 12a) [130]. The above functional nanoparticles were obtained based on the chemical reaction of plant polyphenols with organic monomers. Impressively, metal ions can also participate in the construction of plant polyphenol-derived nanoparticles.

In work reported by Liu et al., novel biodegradable EGCG-Fe^3+^/PVP (EFPP) nanoparticles possessing ultra-small size and ideal biocompatibility were prepared based on the complexation effect of EGCG, Fe^3+^ ions, and PVP (Figure 12b), which showed good suppression of Aβ40 fibrillation and can also decompose existing Aβ40 fibrils owing to the weak hydrophobicity of PVP and antioxidant activity from EGCG [131]. The obtained EFPP nanoparticles had great application potential in the treatment of Alzheimer’s disease. Similarly, Zeng et al. fabricated novel pH-sensitive Fe^3+^-gallic acid nanoparticles, which exhibited strong NIR absorbance. The size of the nanoparticles can be controlled by adjusting the solution pH value. The obtained nanoparticles were unstable under neutral conditions but stable under mild acidic conditions (pH ~5.0), which can be used for cancer diagnosis and treatment. In addition, due to the excellent photothermal effect and low toxicity, Fe^3+^-gallic acid nanoparticles had the potential as a biomedical photothermal agent [132]. In addition, Wang et al. synthesized a series of Fe^3+^-phenol nanoparticles based on plant polyphenols having different structures through a facile one-step complex method. The results showed that the prepared Fe^3+^-pyrocatechol and Fe^3+^-tannic acid nanoparticles possessed the desired photothermal conversion capacity and can further lead to MCF-7 cell death under laser irradiation, as well as exhibiting enormous potential as photoacoustic agents [133]. To date, various polyphenol-based nanoparticles can be designed and prepared via intra- and intermolecular interactions of plant polyphenols. In addition, the morphology and size of polyphenol-based nanoparticles with or without other macromolecules/metal ions can also be regulated by these diverse and reversible interactions, including self-assembly, polymerization, and coordination. Benefiting from their good biocompatibility, polyphenol-based nanoparticles have shown great advantages in medical fields, such as drug delivery, magnetic resonance imaging, photoacoustic imaging, and so on.

As mentioned before, the abundant catechol groups in plant polyphenols can chelate with different types of metal ions to achieve the construction of multifunctional nanoparticles. Importantly, the primary nanoparticles can be first prepared based on small-molecular plant polyphenols through a variety of intermolecular interactions (including host–guest interaction, hydrophobic interaction, self-polymerization, etc.), which are rich in Ph-OH groups. Therefore, the secondary structured nanoparticles can be further constructed through metal ion modification on their basis. Altogether, the multi-level polymerization/self-assembly strategy is of great significance for the in-depth understanding of the assembly process from the molecular to nano (or macro) scales and also provides a reliable method for the construction of advanced smart materials.

### 3.8. Other Functional Materials

In addition to the above methods for preparing plant polyphenol-based materials, other pathways of obtaining multifunctional biobased materials with fascinating applications in the food, environmental, and biomedical fields have also been reported [134,135,136,137].

For instance, in the work published by Yuan et al., an active edible film was developed by introducing tea polyphenols into calcium alginate gel. The results showed that with an increase in the tea polyphenol amounts, the mechanical properties and water vapor permeability were improved. Additionally, after introducing tea polyphenols, the target film exhibited enhanced antioxidant capacity and anti-inflammatory properties, which had great potential in the food packaging field [138]. In addition, Yin and co-workers fabricated an active and intelligent collagen-based packaging film with collagen, delphinidin (polyphenolic compound), and laccase, which showed good dry/wet tensile strengths (41.74 MPa and 13.13 MPa), antioxidant ability, and barrier properties. Benefiting from the good antioxidant activity of the polyphenolic compound, the packaging film can extend the shelf life of food. Importantly, after further incorporating vaccinium oxycoccus pigment (another type of polyphenolic compound), the target packaging film can be used to monitor the freshness of protein/fat-rich food through color changes, owing to the different structures of vaccinium oxycoccus pigments at different pH conditions (pH-sensing indicator) [139]. Consequently, based on the antioxidant capacity and pH responsiveness of polyphenolic compounds, the plant polyphenol-derived functional films will show great application potential in the field of smart food packaging. Furthermore, regarding the environment, Deng et al. reported a new nano-sized adsorbent for Pb^2+^ based on the polyphenolic condensation of tea polyphenols, which showed a relatively high removal efficiency (>90%, 584.8 mg/g at pH 5.5 and 25 °C) due to the presence of abundant Ph-OH groups [140]. The method in this work demonstrated that the tea polyphenols can be further applied in the removal of heavy metal ions from wastewater.

**Figure 13:**
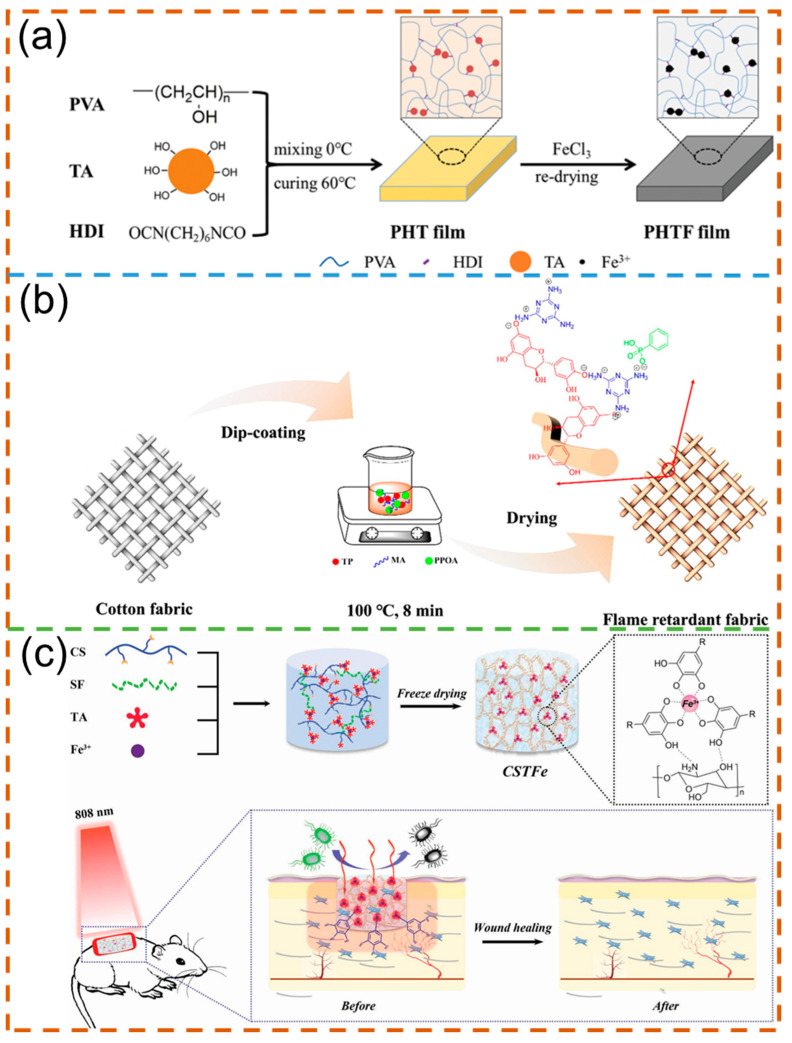
(**a**) Schematic of the preparation of the poly(vinyl alcohol) shape memory polymer. Reprinted from Reference [138] with permission of Royal Society of Chemistry, Copyrights (2022). (**b**) Schematic of the preparation of the flame retardant fabric. Reprinted from Reference [139] with permission of Elsevier, Copyrights (2022). (**c**) Schematic of the preparation of the cryogel and its application as a wound dressing. Reprinted from Reference [140] with permission of John Wiley and Sons, Copyrights (2019).

In terms of plant polyphenol-derived functional polymers, Bai et al. prepared a new type of triple-responsive shape memory polymer based on the crosslinked poly(vinyl alcohol) and tannic acid/Fe^3+^ complex (Figure 13a). In this work, benefiting from the hydrophilic poly(vinyl alcohol) and excellent photothermal conversion capability of the tannic acid/Fe^3+^ complex, the resultant polymer can achieve good water-, thermal-, and NIR light-triggered shape memory properties, where the shape recovery ratios triggered by the above three stimuli reached over 95% [141]. Plant polyphenols can also be used for fabric finishing. For example, Jiang et al. fabricated a new phosphorus–nitrogen flame retardant on the basis of tea polyphenol, melamine, and phenylphosphonic acid (Figure 13b), which can generate free radicals and non-flammable gases (such as N_2_ and NH_3_) during combustion. The results showed that the cotton fabrics coated with the flame retardant possessed good flame retardancy (the LOI value reached to 28.7%, the peak heat release rate was reduced by 88.5%) and UV resistance [142]. The method in this study provided a good alternative for the preparation of a green flame retardant. In addition, plant polyphenols can be further applied in the field of biomedicine. In work reported by Yu et al., a low-cost biobased cryogel was successfully prepared by using tannic acid/Fe^3+^ as the photothermal agent and chitosan-silk fibroin as the skeleton (Figure 13c). Due to the effect of the hydrogen bond and coordination bond, the prepared cryogel showed ideal flexibility and recoverability. Meanwhile, benefiting from the porous structure of the matrix and photothermal conversion ability of the tannic acid/Fe^3+^ complex, the target cryogel can be used as a wound dressing to absorb blood for hemostasis and kill bacteria, as well as promoting skin regeneration [143]. It can be reasonably inferred that plant polyphenol-derived wound dressing will demonstrate greater potential for clinical applications due to their excellent comprehensive performance. In addition, Kohli et al. developed a dental bleaching agent with polyphenols (strawberry extract), which showed ideal bleaching properties and antimicrobial efficacy [144]. Due to the acidic properties of polyphenolic compounds, they can serve as strong oxidizing agents on the surface of tooth enamel.

There are many other applications of plant polyphenols, such as in the fields of drug delivery [145,146,147], nanocoating [148,149,150], cancer treatment [151,152,153], etc. The latest works will be further systematically summarized and discussed in future reviews. In a word, due to the high chemical reactivity and unique properties of biobased plant polyphenols, they have been widely utilized in various multifunctional polymeric materials.

## 4. Conclusions and Outlook

Nowadays, many commercial chemicals/products are derived from fossil feedstocks. On account of the environmental concerns and increasing consumption of fossil resources, more and more scientists’ attention is being drawn to the exploration of biobased materials. Plant polyphenols are biobased resources with unique features that can be obtained from various plants, which have not yet been fully exploited as green and sustainable components to prepare novel polymeric materials. The diverse structural derivatives, ease of processing, and high physical or/and chemical reactivities of plant polyphenols allow them to undergo different modifications (such as Mannich reaction, coupling reaction, coordination reaction, etc.), giving the opportunity to construct biobased materials with comparable, improved, or even extended properties by using plant polyphenols as bio-sourced alternatives. Moreover, pure or modified plant polyphenols permit the creation of polyurethanes, epoxy resins, leathers, hydrogels, nanoparticles, etc., with interesting properties and are able to overcome or alleviate the inherent deficiencies that cannot be solved by each separate component in a system. Significantly, whether introducing plant polyphenols through surface/interface modification, in situ polymerization, or physical blending, the resultant products will obtain multifunctionality or secondary reaction activity, making plant polyphenols potential candidates to meet the needs of current polymers.

To date, plant polyphenols have become one of the most important resource treasures being utilized by humans. All of the current progress highlights the enormous potential of plant polyphenols in creating innovative and high-performance materials, in line with the emerging concept of green and sustainable development. The application fields of plant polyphenol-based polymeric materials will also be easily expanded through reasonable structural design combined with the most promising areas currently available. However, it should be noted that there are some limitations associated with the intrinsic properties of plant polyphenols that need to be resolved. For instance, due to the diversity of plant polyphenols, the extraction processes are not easily reproducible, and their applications in fine chemicals are relatively difficult to achieve. In addition, owing to the influence of the polymerization degree and chemical branching structures of plant polyphenols, the development of polyphenol derivatives may be restricted to some extent. In addition, for some biobased polyphenolic materials, the proportion of plant polyphenols in the system is relatively low. Importantly, many of the above reported works are still at the laboratory scale, and, thus, the industrial application research of plant polyphenols needs to be further strengthened. The effective extraction of plant polyphenols is the premise of their application. The plant polyphenol extraction methods are now more focused on the extraction yield and efficiency, but the accompanying environmental impacts should not be ignored. In the coming years, more attention should also be paid to the scaling up and economic analysis of plant polyphenol extraction processes. More importantly, in order to obtain high-value-added plant polyphenol products, in-depth studies about the various chemical structures of plant polyphenols and the utilization of refined plant polyphenols (and derivatives) are a priority. Meanwhile, controlling the polymerization processes (e.g., structural design, reaction condition, and polymerization degree) of polyphenolic compounds is another key area in developing high-performance plant polyphenol-based polymeric materials. Fortunately, more and more researchers and industry peers are focusing on and studying plant polyphenol chemistry, joining in the design and elaboration of plant polyphenol-based polymers and products. We believe that plant polyphenols will be able to be applied in real applications in the near future. Altogether, biobased polymers represent an important research area in green chemistry, and plant polyphenols are emerging as an encouraging and fascinating renewable bioresource to replace fossil derivatives.

## References

[1] Hernes P.J., Hedges J.I. (2004). Tannin signatures of barks, needles, leaves, cones, and wood at the molecular level11Associate editor: C. Arnosti. Geochim. Cosmochim. Acta.

[2] Hu Q., Luo Y. (2016). Polyphenol-chitosan conjugates: Synthesis, characterization, and applications. Carbohydr. Polym..

[3] Zagoskina N.V., Zubova M.Y., Nechaeva T.L., Kazantseva V.V., Goncharuk E.A., Katanskaya V.M., Baranova E.N., Aksenova M.A. (2023). Polyphenols in Plants: Structure, Biosynthesis, Abiotic Stress Regulation, and Practical Applications (Review). Int. J. Mol. Sci..

[4] Li L., Zhao J., Yang T., Sun B. (2022). High-speed countercurrent chromatography as an efficient technique for large separation of plant polyphenols: A review. Food Res. Int..

[5] Vera M., Silva C., Li N.-N., García Y., Jiménez V.A., Urbano B.F. (2024). Laccase-mediated polymerization of tannins from a pine bark extract: Toward an eco-friendly valorization of forest wastes. J. Appl. Polym. Sci..

[6] Mueller-Harvey I. (2001). Analysis of hydrolysable tannins. Anim. Feed Sci. Technol..

[7] Dzah C.S., Duan Y., Zhang H., Serwah Boateng N.A., Ma H. (2020). Latest developments in polyphenol recovery and purification from plant by-products: A review. Trends Food Sci. Technol..

[8] Tondi G., Thevenon M.F., Mies B., Standfest G., Petutschnigg A., Wieland S. (2013). Impregnation of Scots pine and beech with tannin solutions: Effect of viscosity and wood anatomy in wood infiltration. Wood Sci. Technol..

[9] Fairhead V.A., Amsler C.D., McClintock J.B., Baker B.J. (2005). Variation in phlorotannin content within two species of brown macroalgae (*Desmarestia anceps* and *D. menziesii*) from the Western Antarctic Peninsula. Polar Biol..

[10] Schoenwaelder M.E.A. (2002). The occurrence and cellular significance of physodes in brown algae. Phycologia.

[11] Arbenz A., Avérous L. (2015). Chemical modification of tannins to elaborate aromatic biobased macromolecular architectures. Green Chem..

[12] Bacelo H.A.M., Santos S.C.R., Botelho C.M.S. (2016). Tannin-based biosorbents for environmental applications-A review. Chem. Eng. J..

[13] de Hoyos-Martínez P.L., Merle J., Labidi J., Charrier-El Bouhtoury F. (2019). Tannins extraction: A key point for their valorization and cleaner production. J. Clean. Prod..

[14] Ebrahimnezhad-Khaljiri H., Ghadi A. (2024). Recent advancement in synthesizing bio-epoxy nanocomposites using lignin, plant oils, saccharides, polyphenols, and natural rubbers: A review. Int. J. Biol. Macromol..

[15] Vera M., Urbano B.F. (2021). Tannin polymerization: An overview. Polym. Chem..

[16] Falcão L., Araújo M.E.M. (2018). Vegetable Tannins Used in the Manufacture of Historic Leathers. Molecules.

[17] Qiao D.-W., Yao J., Song L.-J., Yang J.-Y. (2021). Migration of leather tannins and chromium in soils under the effect of simulated rain. Chemosphere.

[18] Vuthiganond N., Nakpathom M., Mongkholrattanasit R. (2020). Azoic Deep Dyeing of Silk and UV Protection Using Plant Polyphenols and Diazonium Coupling. Fibers Polym..

[19] Lund M.N. (2021). Reactions of plant polyphenols in foods: Impact of molecular structure. Trends Food Sci. Technol..

[20] Senusi F., Nasuha N., Husain A., Ismail S. (2022). Synthesis of catechol-amine coating solution for membrane surface modification. Environ. Sci. Pollut. Res..

[21] Feng Y.-Y., Chen Y.-Q., Wang Z., Wei J. (2022). Synthesis of mesoporous carbon materials from renewable plant polyphenols for environmental and energy applications. New Carbon Mater..

[22] Zhao X., Yan F., Li X.-S., Qu D., Xu Y.-L. (2023). A systematic review of tea pigments: Prevention of major diseases, protection of organs, and potential mechanisms and applications. Food Sci. Nutr..

[23] Peng H., Du X., Cheng X., Wang H., Du Z. (2021). Room-temperature self-healable and stretchable waterborne polyurethane film fabricated via multiple hydrogen bonds. Prog. Org. Coat..

[24] Zhao Y., Zhang Q., Lei H., Zhou X., Du G., Pizzi A., Xi X. (2024). Preparation and fire resistance modification on tannin-based non-isocyanate polyurethane (NIPU) rigid foams. Int. J. Biol. Macromol..

[25] Ren L., Ma X., Zhang J., Qiang T. (2020). Preparation of gallic acid modified waterborne polyurethane made from bio-based polyol. Polymer.

[26] Wan J., Zhao J., Zhang X., Fan H., Zhang J., Hu D., Jin P., Wang D.-Y. (2020). Epoxy thermosets and materials derived from bio-based monomeric phenols: Transformations and performances. Prog. Polym. Sci..

[27] Ristić M., Samaržija-Jovanović S., Jovanović V., Kostić M., Erceg T., Jovanović T., Marković G., Marinović-Cincović M. (2023). Hydrolytic and thermal stability of urea-formaldehyde resins based on tannin and betaine bio-fillers. J. Vinyl Addit. Technol..

[28] Santiago-Medina F.-J., Pizzi A., Basso M.C., Delmotte L., Celzard A. (2017). Polycondensation Resins by Flavonoid Tannins Reaction with Amines. Polymers.

[29] Wang J., Zhou S.-Y., Qu Y., Yang B., Zhang Q., Lin Y., Lu G.-P. (2023). Modified Gallic Acids as Both Reactive Flame Retardants and Cross-Linkers for the Fabrication of Flame-Retardant Polyurethane Elastomers. ChemistrySelect.

[30] Wen Y., Wang J., Wang F., Wu H., Zhou J., Dai Z., Guo H. (2024). Recent advances in membranes modified with plant polyphenols in wastewater treatment: A review. Sep. Purif. Technol..

[31] Meng X., Chen L., Lv R., Liu M., He N., Wang Z. (2020). A metal–phenolic network-based multifunctional nanocomposite with pH-responsive ROS generation and drug release for synergistic chemodynamic/photothermal/chemo-therapy. J. Mater. Chem. B.

[32] Quideau S., Deffieux D., Douat-Casassus C., Pouységu L. (2011). Plant Polyphenols: Chemical Properties, Biological Activities, and Synthesis. Angew. Chem. Int. Ed..

[33] Pimentel-Moral S., Teixeira M.C., Fernandes A.R., Arráez-Román D., Martínez-Férez A., Segura-Carretero A., Souto E.B. (2018). Lipid nanocarriers for the loading of polyphenols- A comprehensive review. Adv. Colloid Interface Sci..

[34] Renu K., Mukherjee A.G., Gopalakrishnan A.V., Wanjari U.R., Kannampuzha S., Murali R., Veeraraghavan V.P., Vinayagam S., Paz-Montelongo S., George A. (2023). Protective effects of macromolecular polyphenols, metals (zinc, selenium, and copper)—Polyphenol complexes, and different organs with an emphasis on arsenic poisoning: A review. Adv. Colloid Interface Sci..

[35] Shirmohammadli Y., Efhamisisi D., Pizzi A. (2018). Tannins as a sustainable raw material for green chemistry: A review. Ind. Crops Prod..

[36] Khanbabaee K., van Ree T. (2001). Tannins: Classification and Definition. Nat. Prod. Rep..

[37] Koopmann A.-K., Schuster C., Torres-Rodríguez J., Kain S., Pertl-Obermeyer H., Petutschnigg A., Hüsing N. (2020). Tannin-Based Hybrid Materials and Their Applications: A Review. Molecules.

[38] Pizzi A. (2006). Recent developments in eco-efficient bio-based adhesives for wood bonding: Opportunities and issues. J. Adhes. Sci. Technol..

[39] Huang Q., Liu X., Zhao G., Hu T., Wang Y. (2018). Potential and challenges of tannins as an alternative to in-feed antibiotics for farm animal production. Anim. Nutr..

[40] Okuda T. (2005). Systematics and health effects of chemically distinct tannins in medicinal plants. Phytochemistry.

[41] Fraga-Corral M., García-Oliveira P., Pereira A.G., Lourenço-Lopes C., Jimenez-Lopez C., Prieto M.A., Simal-Gandara J. (2020). Technological Application of Tannin-Based Extracts. Molecules.

[42] Zhang C., Xue J., Yang X., Ke Y., Ou R., Wang Y., Madbouly S.A., Wang Q. (2022). From plant phenols to novel bio-based polymers. Prog. Polym. Sci..

[43] Schofield P., Mbugua D.M., Pell A.N. (2001). Analysis of condensed tannins: A review. Anim. Feed Sci. Technol..

[44] Shi L., Zhang Y., Tong Y., Ding W., Li W. (2023). Plant-inspired biomimetic hybrid PVDF membrane co-deposited by tea polyphenols and 3-amino-propyl-triethoxysilane for high-efficiency oil-in-water emulsion separation. J. Chem. Eng..

[45] Xiao S., Wei J., Jin S., Xia X., Yuan L., Zou Q., Zuo Y., Li J., Li Y. (2023). A Multifunctional Coating Strategy for Promotion of Immunomodulatory and Osteo/Angio-Genic Activity. Adv. Funct. Mater..

[46] Nicollin A., Zhou X., Pizzi A., Grigsby W., Rode K., Delmotte L. (2013). MALDI-TOF and 13C NMR analysis of a renewable resource additive-Thermoplastic acetylated tannins. Ind. Crops Prod..

[47] Braghiroli F.L., Fierro V., Izquierdo M.T., Parmentier J., Pizzi A., Celzard A. (2012). Nitrogen-doped carbon materials produced from hydrothermally treated tannin. Carbon.

[48] Hashida K., Makino R., Ohara S. (2009). Amination of pyrogallol nucleus of condensed tannins and related polyphenols by ammonia water treatment. Holzforschung.

[49] Braghiroli F., Fierro V., Pizzi A., Rode K., Radke W., Delmotte L., Parmentier J., Celzard A. (2013). Reaction of condensed tannins with ammonia. Ind. Crops Prod..

[50] Soto R., Freer J., Baeza J. (2005). Evidence of chemical reactions between di- and poly-glycidyl ether resins and tannins isolated from Pinus radiata D. Don bark. Bioresour. Technol..

[51] Luo S., Yang K., Zhong Z., Wu X., Ren T. (2018). Facile preparation of degradable multi-arm-star-branched waterborne polyurethane with bio-based tannic acid. RSC Adv..

[52] Mira L., Tereza Fernandez M., Santos M., Rocha R., Helena Florêncio M., Jennings K.R. (2002). Interactions of Flavonoids with Iron and Copper Ions: A Mechanism for their Antioxidant Activity. Free Radic. Res..

[53] Zhang L., Guan Q., Jiang J., Khan M.S. (2023). Tannin complexation with metal ions and its implication on human health, environment and industry: An overview. Int. J. Biol. Macromol..

[54] Gao M., Wang Z., Yang C., Ning J., Zhou Z., Li G. (2019). Novel magnetic graphene oxide decorated with persimmon tannins for efficient adsorption of malachite green from aqueous solutions. Colloids Surf. A.

[55] Cao H., Yang L., Tian R., Wu H., Gu Z., Li Y. (2022). Versatile polyphenolic platforms in regulating cell biology. Chem. Soc. Rev..

[56] Yin X., Huang Z., Liu X., Sun Y., Lin X., Lin W., Yi G. (2024). Room-temperature self-healing polyurethanes with high mechanical strength and superior toughness for sensor application. J. Appl. Polym. Sci..

[57] Li X.-C., Bi Y.-S., Bi G.-H. (2024). A comparative study of polyurethane foam by substituting LBA using green polyurethane foam CFA-1. J. Appl. Polym. Sci..

[58] Seydibeyoǧlu M.Ö., İşçi S., Güngör N., Ece O.I., Güner F.S. (2010). Preparation of polyurethane/hectorite, polyurethane/montmorillonite, and polyurethane/laponite nanocomposites without organic modifiers. J. Appl. Polym. Sci..

[59] Gogoi S., Karak N. (2014). Biobased Biodegradable Waterborne Hyperbranched Polyurethane as an Ecofriendly Sustainable Material. ACS Sustain. Chem. Eng..

[60] Luo S., Fan L., Yang K., Zhong Z., Wu X., Ren T. (2018). In situ and controllable synthesis of Ag NPs in tannic acid-based hyperbranched waterborne polyurethanes to prepare antibacterial polyurethanes/Ag NPs composites. RSC Adv..

[61] Liu Y., Zhang Z., Wang J., Xie T., Sun L., Yang K., Li Z. (2021). Renewable tannic acid based self-healing polyurethane with dynamic phenol-carbamate network: Simultaneously showing robust mechanical properties, reprocessing ability and shape memory. Polymer.

[62] Liu Y., Li Z., Zhang Z., Wang J., Sun L., Xie T. (2021). Thermal-driven self-healing waterborne polyurethane with robust mechanical properties based on reversible phenol-carbamate network and Fe^3+^-catechol coordination bond. Prog. Org. Coat..

[63] Liu Y., Zhang Z., Fan W., Yang K., Li Z. (2022). Preparation of renewable gallic acid-based self-healing waterborne polyurethane with dynamic phenol-carbamate network: Toward superior mechanical properties and shape memory function. J. Mater. Sci..

[64] Liu Y., Zhang Z., Yang K., Chen D., Li Z. (2022). Novel near-infrared light-induced shape memory nonionic waterborne polyurethane composites based on iron gallate and dynamic phenol-carbamate network. Polymer.

[65] Fang Y., Xu J., Gao F., Du X., Du Z., Cheng X., Wang H. (2021). Self-healable and recyclable polyurethane-polyaniline hydrogel toward flexible strain sensor. Compos. Part B.

[66] Dalton E., Morris Z., Ayres N. (2022). Synthesis and characterization of sulfated-lactose polyurethane hydrogels. Polym. Chem..

[67] Divakaran A.V., Azad L.B., Surwase S.S., Torris A. T. A., Badiger M.V. (2016). Mechanically Tunable Curcumin Incorporated Polyurethane Hydrogels as Potential Biomaterials. Chem. Mater..

[68] Wen J., Pan M., Yuan J., Wang J., Zhu L., Jia Z., Song S. (2020). Facile fabrication of dual-crosslinked single network heterostructural polyurethane hydrogels with superior mechanical and fluorescent performance. React. Funct. Polym..

[69] Liu Y., Zhang Z., Liang Z., Yong Y., Yang C., Li Z. (2022). Multifunctional polyurethane hydrogel based on a phenol–carbamate network and an Fe^3+^–polyphenol coordination bond toward NIR light triggered actuators and strain sensors. J. Mater. Chem. A.

[70] Arbenz A., Frache A., Cuttica F., Avérous L. (2016). Advanced biobased and rigid foams, based on urethane-modified isocyanurate from oxypropylated gambier tannin polyol. Polym. Degrad. Stab..

[71] Borrero-López A.M., Nicolas V., Marie Z., Celzard A., Fierro V. (2022). A Review of Rigid Polymeric Cellular Foams and Their Greener Tannin-Based Alternatives. Polymers.

[72] Ge J., Shi X., Cai M., Wu R., Wang M. (2003). A novel biodegradable antimicrobial PU foam from wattle tannin. J. Appl. Polym. Sci..

[73] Oliviero M., Stanzione M., D’Auria M., Sorrentino L., Iannace S., Verdolotti L. (2019). Vegetable Tannin as a Sustainable UV Stabilizer for Polyurethane Foams. Polymers.

[74] Yang Z., Guo W., Yang P., Hu J., Duan G., Liu X., Gu Z., Li Y. (2021). Metal-phenolic network green flame retardants. Polymer.

[75] Zeng F.-R., Liu B.-W., Wang Z.-H., Zhang J.-Y., Chen X.-L., Zhao H.-B., Wang Y.-Z. (2023). Recyclable Biophenolic Nanospheres for Sustainable and Durable Multifunctional Applications in Thermosets. ACS Mater. Lett..

[76] Guan J., Song Y., Lin Y., Yin X., Zuo M., Zhao Y., Tao X., Zheng Q. (2011). Progress in Study of Non-Isocyanate Polyurethane. Ind. Eng. Chem. Res..

[77] Thébault M., Pizzi A., Dumarçay S., Gerardin P., Fredon E., Delmotte L. (2014). Polyurethanes from hydrolysable tannins obtained without using isocyanates. Ind. Crops Prod..

[78] Chen X., Li J., Xi X., Pizzi A., Zhou X., Fredon E., Du G., Gerardin C. (2020). Condensed tannin-glucose-based NIPU bio-foams of improved fire retardancy. Polym. Degrad. Stab..

[79] Sternberg J., Sequerth O., Pilla S. (2021). Green chemistry design in polymers derived from lignin: Review and perspective. Prog. Polym. Sci..

[80] Xia L., Wang X., Ren T., Luo L., Li D., Dai J., Xu Y., Yuan C., Zeng B., Dai L. (2022). Green construction of multi-functional fire resistant epoxy resins based on boron nitride with core-shell structure. Polym. Degrad. Stab..

[81] Yang Y., Wang D.-Y., Jian R.-K., Liu Z., Huang G. (2023). Chemical structure construction of DOPO-containing compounds for flame retardancy of epoxy resin: A review. Prog. Org. Coat..

[82] Zhang K., Wang Y., Chen Y., Li W., Chang Q., He Z., Zhu Y., Huang J., Nie X. (2024). Construction of multiple sacrificial bonds between epoxy resin and tung oil-based modifier towards mechanical performance enhancement. Ind. Crops Prod..

[83] Rad E.R., Vahabi H., de Anda A.R., Saeb M.R., Thomas S. (2019). Bio-epoxy resins with inherent flame retardancy. Prog. Org. Coat..

[84] Kumar S., Krishnan S., Mohanty S., Nayak S.K. (2018). Synthesis and characterization of petroleum and biobased epoxy resins: A review. Polym. Int..

[85] Capricho J.C., Fox B., Hameed N. (2020). Multifunctionality in Epoxy Resins. Polym. Rev..

[86] Gonçalves F.A.M.M., Ferreira P., Alves P. (2021). Synthesis and characterization of itaconic-based epoxy resin: Chemical and thermal properties of partially biobased epoxy resins. Polymer.

[87] Liang D., Yang J., Li X., Wang W., Qi J., Lu Z. (2024). Preparation and physical properties of CeO_2_ doped and modified epoxy resin composites. Polym. Compos..

[88] Brand S., Veith L., Baier R., Dietrich C., Schmid M.J., Ternes T.A. (2020). New methodical approaches for the investigation of weathered epoxy resins used for corrosion protection of steel constructions. J. Hazard. Mater..

[89] Benyahya S., Aouf C., Caillol S., Boutevin B., Pascault J.P., Fulcrand H. (2014). Functionalized green tea tannins as phenolic prepolymers for bio-based epoxy resins. Ind. Crops Prod..

[90] Long Y., Han W., Xing Z., Hao C. (2024). Synthesis of pyrogallol triglycidyl ether: A bio-based epoxy resin monomer with low viscosity, high activity, and good thermomechanical properties. Polymer.

[91] Esmaeili N., Salimi A., Zohuriaan-Mehr M.J., Vafayan M., Meyer W. (2018). Bio-based thermosetting epoxy foam: Tannic acid valorization toward dye-decontaminating and thermo-protecting applications. J. Hazard. Mater..

[92] Kim Y.-O., Cho J., Yeo H., Lee B.W., Moon B.J., Ha Y.-M., Jo Y.R., Jung Y.C. (2019). Flame Retardant Epoxy Derived from Tannic Acid as Biobased Hardener. ACS Sustain. Chem. Eng..

[93] Feng X., Fan J., Li A., Li G. (2020). Biobased Tannic Acid Cross-Linked Epoxy Thermosets with Hierarchical Molecular Structure and Tunable Properties: Damping, Shape Memory, and Recyclability. ACS Sustain. Chem. Eng..

[94] Xu Y., Guo L., Zhang H., Zhai H., Ren H. (2019). Research status, industrial application demand and prospects of phenolic resin. RSC Adv..

[95] Li J., Zhu W., Zhang S., Gao Q., Xia C., Zhang W., Li J. (2019). Depolymerization and characterization of Acacia mangium tannin for the preparation of mussel-inspired fast-curing tannin-based phenolic resins. Chem. Eng. J..

[96] Hafiz N.L.M., Tahir P.M., Hua L.S., Abidin Z.Z., Sabaruddin F.A., Yunus N.M., Abdullah U.H., Abdul Khalil H.P.S. (2020). Curing and thermal properties of co-polymerized tannin phenol-formaldehyde resin for bonding wood veneers. J. Mater. Res. Technol..

[97] Santiago-Medina F., Foyer G., Pizzi A., Caillol S., Delmotte L. (2016). Lignin-derived non-toxic aldehydes for ecofriendly tannin adhesives for wood panels. Int. J. Adhes. Adhes..

[98] Lacoste C., Basso M.C., Pizzi A., Laborie M.P., Garcia D., Celzard A. (2013). Bioresourced pine tannin/furanic foams with glyoxal and glutaraldehyde. Ind. Crops Prod..

[99] Szczurek A., Fierro V., Pizzi A., Stauber M., Celzard A. (2013). Carbon meringues derived from flavonoid tannins. Carbon.

[100] Grigsby W.J., Bridson J.H., Lomas C., Elliot J.-A. (2013). Esterification of Condensed Tannins and Their Impact on the Properties of Poly(Lactic Acid). Polymers.

[101] Xia Z., Kiratitanavit W., Facendola P., Thota S., Yu S., Kumar J., Mosurkal R., Nagarajan R. (2018). Fire resistant polyphenols based on chemical modification of bio-derived tannic acid. Polym. Degrad. Stab..

[102] Song P., Jiang S., Ren Y., Zhang X., Qiao T., Song X., Liu Q., Chen X. (2016). Synthesis and characterization of tannin grafted polycaprolactone. J. Colloid Interface Sci..

[103] Goto T., Iwata T., Abe H. (2019). Synthesis and Characterization of Biobased Polyesters Containing Anthraquinones Derived from Gallic Acid. Biomacromolecules.

[104] Gazzotti S., Hakkarainen M., Adolfsson K.H., Ortenzi M.A., Farina H., Lesma G., Silvani A. (2018). One-Pot Synthesis of Sustainable High-Performance Thermoset by Exploiting Eugenol Functionalized 1,3-Dioxolan-4-one. ACS Sustain. Chem. Eng..

[105] Teklemedhin T.B., Gebretsadik T.T., Gebrehiwet T.B., Gebrekidan G.A., Edris M., Teklegiorgis N.T., Hagos K.B. (2023). Vegetable Tannins as Chrome-Free Leather Tanning. Adv. Mater. Sci. Eng..

[106] Xiao Y., Zhou J., Wang C., Zhang J., Radnaeva V.D., Lin W. (2023). Sustainable metal-free leather manufacture via synergistic effects of triazine derivative and vegetable tannins. Collagen Leather.

[107] Zhang Z., Liu Y., Wang J., Xie T., Sun L., Li Z. (2021). A chrome-free combination tanning strategy: Based on silicic acid and plant tannin. J. Leather Sci. Eng..

[108] Marsal A., Cuadros S., Manich A.M., Izquierdo F., Font J. (2017). Reduction of the formaldehyde content in leathers treated with formaldehyde resins by means of plant polyphenols. J. Clean. Prod..

[109] Yu R., Wang H., Wang R., Zhao P., Chen Y., Liu G., Liao X. (2022). Polyphenol modified natural collagen fibrous network towards sustainable and antibacterial microfiltration membrane for efficient water disinfection. Water Res..

[110] Yan L., Zhou J., Li H., Zhong R., Zhuang J., Xu X., Wang Y., Liao X., Shi B. (2023). Wearable synthetic leather-based high-performance X-ray shielding materials enabled by the plant polyphenol- and hierarchical structure-facilitated dispersion. Collagen Leather.

[111] Bahrami Z., Akbari A., Eftekhari-Sis B. (2019). Double network hydrogel of sodium alginate/polyacrylamide cross-linked with POSS: Swelling, dye removal and mechanical properties. Int. J. Biol. Macromol..

[112] Ahmed E.M. (2015). Hydrogel: Preparation, characterization, and applications: A review. J. Adv. Res..

[113] Sun Z., Song C., Zhou J., Hao C., Liu W., Liu H., Wang J., Huang M., He S., Yang M. (2021). Rapid Photothermal Responsive Conductive MXene Nanocomposite Hydrogels for Soft Manipulators and Sensitive Strain Sensors. Macromol. Rapid Commun..

[114] Yu J., Feng Y., Sun D., Ren W., Shao C., Sun R. (2022). Highly Conductive and Mechanically Robust Cellulose Nanocomposite Hydrogels with Antifreezing and Antidehydration Performances for Flexible Humidity Sensors. ACS Appl. Mater. Interfaces.

[115] Wei X., Lin T., Gao J., Hu Y., Zhang Z., Peng J., Li J., Zhai M. (2024). Mechanically Robust and Highly Conductive Poly(ionic liquid)/Polyacrylamide Double-Network Hydrogel Electrolytes for Flexible Symmetric Supercapacitors with a Wide Operating Voltage Range. ACS Appl. Mater. Interfaces.

[116] Li X., Zhao X., Liu R., Wang H., Wang S., Fan B., Hu C., Wang H. (2024). Mussel-inspired PDA@PEDOT nanocomposite hydrogel with excellent mechanical strength, self-adhesive, and self-healing properties for a flexible strain sensor. J. Mater. Chem. B.

[117] Zhang J., Yan K., Huang J., Sun X., Li J., Cheng Y., Sun Y., Shi Y., Pan L. (2024). Mechanically Robust, Flexible, Fast Responding Temperature Sensor and High-Resolution Array with Ionically Conductive Double Cross-Linked Hydrogel. Adv. Funct. Mater..

[118] Sun Z., Wei C., Liu W., Liu H., Liu J., Hao R., Huang M., He S. (2021). Two-Dimensional MoO_2_ Nanosheet Composite Hydrogels with High Transmittance and Excellent Photothermal Property for Near-Infrared Responsive Actuators and Microvalves. ACS Appl. Mater. Interfaces.

[119] Yin B., Gosecka M., Bodaghi M., Crespy D., Youssef G., Dodda J.M., Wong S.H.D., Imran A.B., Gosecki M., Jobdeedamrong A. (2024). Engineering multifunctional dynamic hydrogel for biomedical and tissue regenerative applications. Chem. Eng. J..

[120] Zhang L., Shen Q., Cheng Y.-F. (2022). Fabrication, characterization and application of chitosan/tea polyphenols blending hydrogels. Bull. Mater. Sci..

[121] Kim B.S., Kim S.H., Kim K., An Y.H., So K.H., Kim B.G., Hwang N.S. (2020). Enzyme-mediated one-pot synthesis of hydrogel with the polyphenol cross-linker for skin regeneration. Mater. Today Bio.

[122] Deng H., Yu Z., Chen S., Fei L., Sha Q., Zhou N., Chen Z., Xu C. (2020). Facile and eco-friendly fabrication of polysaccharides-based nanocomposite hydrogel for photothermal treatment of wound infection. Carbohydr. Polym..

[123] Fan X., Zhao L., Ling Q., Gu H. (2022). Tough, Self-Adhesive, Antibacterial, and Recyclable Supramolecular Double Network Flexible Hydrogel Sensor Based on PVA/Chitosan/Cyclodextrin. Ind. Eng. Chem. Res..

[124] Liu J., Bao S., Ling Q., Fan X., Gu H. (2022). Ultra-fast preparation of multifunctional conductive hydrogels with high mechanical strength, self-healing and self-adhesive properties based on Tara Tannin-Fe^3+^ dynamic redox system for strain sensors applications. Polymer.

[125] Zhang X., Chen L., Zhang C., Liao L. (2021). Robust Near-Infrared-Responsive Composite Hydrogel Actuator Using Fe^3+^/Tannic Acid as the Photothermal Transducer. ACS Appl. Mater. Interfaces.

[126] Liu Y., Zhang Z., Yang X., Li F., Liang Z., Yong Y., Dai S., Li Z. (2023). A stretchable, environmentally stable, and mechanically robust nanocomposite polyurethane organohydrogel with anti-freezing, anti-dehydration, and electromagnetic shielding properties for strain sensors and magnetic actuators. J. Mater. Chem. A.

[127] Zhao Y., Xu L., Kong F., Yu L. (2021). Design and preparation of poly(tannic acid) nanoparticles with intrinsic fluorescence: A sensitive detector of picric acid. Chem. Eng. J..

[128] Yang P., Zhou X., Zhang J., Zhong J., Zhu F., Liu X., Gu Z., Li Y. (2021). Natural polyphenol fluorescent polymer dots. Green Chem..

[129] Han Y., Zhou J., Hu Y., Lin Z., Ma Y., Richardson J.J., Caruso F. (2020). Polyphenol-Based Nanoparticles for Intracellular Protein Delivery via Competing Supramolecular Interactions. ACS Nano.

[130] Wang Z., Ji S., He F., Cao M., Peng S., Li Y. (2018). One-step transformation of highly hydrophobic membranes into superhydrophilic and underwater superoleophobic ones for high-efficiency separation of oil-in-water emulsions. J. Mater. Chem. A.

[131] Liu Z., Li X., Wu X., Zhu C. (2019). A dual-inhibitor system for the effective antifibrillation of Aβ40 peptides by biodegradable EGCG–Fe(iii)/PVP nanoparticles. J. Mater. Chem. B.

[132] Zeng J., Cheng M., Wang Y., Wen L., Chen L., Li Z., Wu Y., Gao M., Chai Z. (2016). pH-Responsive Fe(III)–Gallic Acid Nanoparticles for In Vivo Photoacoustic-Imaging-Guided Photothermal Therapy. Adv. Healthc. Mater..

[133] Wang Y., Liu F., Yan N., Sheng S., Xu C., Tian H., Chen X. (2019). Exploration of FeIII-Phenol Complexes for Photothermal Therapy and Photoacoustic Imaging. ACS Biomater. Sci. Eng..

[134] Pan T.G., Wu Y.N., He S., Wu Z., Jin R. (2022). Food allergenic protein conjugation with plant polyphenols for allergenicity reduction. Curr. Opin. Food Sci..

[135] Feng Y., Li P., Wei J. (2022). Engineering functional mesoporous materials from plant polyphenol based coordination polymers. Coord. Chem. Rev..

[136] Chen Y.-N., Peng L., Liu T., Wang Y., Shi S., Wang H. (2016). Poly(vinyl alcohol)-Tannic Acid Hydrogels with Excellent Mechanical Properties and Shape Memory Behaviors. ACS Appl. Mater. Interfaces.

[137] Zhou L., Fan L., Yi X., Zhou Z., Liu C., Fu R., Dai C., Wang Z., Chen X., Yu P. (2018). Soft Conducting Polymer Hydrogels Cross-Linked and Doped by Tannic Acid for Spinal Cord Injury Repair. ACS Nano.

[138] Biao Y., Yuxuan C., Qi T., Ziqi Y., Yourong Z., McClements D.J., Chongjiang C. (2019). Enhanced performance and functionality of active edible films by incorporating tea polyphenols into thin calcium alginate hydrogels. Food Hydrocoll..

[139] Yin S., Zhang Y., Zhang X., Tao K., Li G. (2023). High-strength collagen/delphinidin film incorporated with Vaccinium oxycoccus pigment for active and intelligent food packaging. Collagen Leather.

[140] Deng Z., Yi Z., Chen G., Ma X., Tang Y., Li X. (2021). Green tea polyphenol nanoparticle as a novel adsorbent to remove Pb^2+^ from wastewater. Mater. Lett..

[141] Bai Y., Ionov L. (2022). A thermo-, near-infrared light- and water-induced shape memory polymer with healing fatigued shape memory performance. Mater. Chem. Front..

[142] Jiang Q., Li P., Liu Y., Zhu P. (2023). Flame retardant cotton fabrics with anti-UV properties based on tea polyphenol-melamine-phenylphosphonic acid. J. Colloid Interface Sci..

[143] Yu Y., Li P., Zhu C., Ning N., Zhang S., Vancso G.J. (2019). Multifunctional and Recyclable Photothermally Responsive Cryogels as Efficient Platforms for Wound Healing. Adv. Funct. Mater..

[144] Kohli S., Bhatia S., Banavar S.R., Al-Haddad A., Kandasamy M., Qasim S.S.B., Kit-Kay M., Pichika M.R., Daood U. (2023). In-vitro evaluation of the effectiveness of polyphenols based strawberry extracts for dental bleaching. Sci. Rep..

[145] Kim K.H., Ki M.-R., Min K.H., Pack S.P. (2023). Advanced Delivery System of Polyphenols for Effective Cancer Prevention and Therapy. Antioxidants.

[146] Chen G., Yi Z., Chen X., Tong Q., Ran Y., Ma L., Li X. (2021). Polymerization-Induced Self-Assembly of Tea Polyphenols into Open-Mouthed Nanoparticles for Active Delivery Systems and Stable Carbon Bowls. ACS Appl. Nano Mater..

[147] Wang X., Fan Y., Yan J., Yang M. (2022). Engineering polyphenol-based polymeric nanoparticles for drug delivery and bioimaging. Chem. Eng. J..

[148] Behboodi-Sadabad F., Li S., Lei W., Liu Y., Sommer T., Friederich P., Sobek C., Messersmith P.B., Levkin P.A. (2021). High-throughput screening of multifunctional nanocoatings based on combinations of polyphenols and catecholamines. Mater. Today Bio.

[149] Centurion F., Hassan M.M., Tang J., Allioux F.-M., Chakraborty S., Chen R., Mao G., Kumar N., Kalantar-Zadeh K., Rahim M.A. (2022). Assembly of surface-independent polyphenol/liquid gallium composite nanocoatings. Nanoscale.

[150] Wang Y., Wang M., Wang Q., Wang T., Zhou Z., Mehling M., Guo T., Zou H., Xiao X., He Y. (2023). Flowthrough Capture of Microplastics through Polyphenol-Mediated Interfacial Interactions on Wood Sawdust. Adv. Mater..

[151] Trisha A.T., Shakil M.H., Talukdar S., Rovina K., Huda N., Zzaman W. (2022). Tea Polyphenols and Their Preventive Measures against Cancer: Current Trends and Directions. Foods.

[152] Farghadani R., Naidu R. (2023). The anticancer mechanism of action of selected polyphenols in triple-negative breast cancer (TNBC). Biomed. Pharmacother..

[153] Zhang L., Kang Q., Kang M., Jiang S., Yang F., Gong J., Ou G., Wang S. (2023). Regulation of main ncRNAs by polyphenols: A novel anticancer therapeutic approach. Phytomedicine.

